# Rapid Bacterial Detection on Surfaces by Field-Deployable Respirometric Sensor Sachets

**DOI:** 10.3390/bios16090503

**Published:** 2026-09-08

**Authors:** Valeria Ferraro, Loris Pinto, Liang Li, Federico Baruzzi, Dmitri B. Papkovsky, Elisa Santovito

**Affiliations:** 1 Institute of Sciences of Food Production, National Research Council of Italy (CNR-ISPA), Via Amendola 122/O, 70125 Bari, Italy; valeriaferraro@cnr.it (V.F.); loris.pinto@cnr.it (L.P.); federico.baruzzi@cnr.it (F.B.); 2 School of Biochemistry and Cell Biology, University College Cork, Cavanagh Pharmacy Building, College Road, T12 YT20 Cork, Ireland; liliang@universityofgalway.ie (L.L.); d.papkovsky@ucc.ie (D.B.P.)

**Keywords:** respirometric biosensor sachets, optical oxygen sensing, on-site microbial detection, surface hygiene monitoring, total viable count (TVC) quantification

## Abstract

Monitoring bacterial contamination on surfaces is critical for hygiene and safety assurance in food, healthcare, and pharmaceutical settings, although routine methods still remain slow and laboratory dependent. Here, we report a portable respirometric platform based on sealed sensor sachets incorporating optical oxygen sensors to rapidly detect and quantify total aerobic viable counts (TVC) from swabbed surfaces. Following standardized surface swabbing, samples were incubated in the sachets and oxygen depletion kinetics were recorded with a handheld reader and microbial activity was inferred from oxygen consumption. Reference quantification was obtained by serial dilution and aerobic plate counting, enabling direct benchmarking of the respirometric readout against an industry-accepted culture method such as ISO 4833:2013. The platform demonstrated strong agreement with plate counts (R^2^ > 0.94), achieving a median detection limit of 2.69 log_10_ CFU/cm^2^ and a dynamic range of 0–6 log_10_ CFU/cm^2^ for surface-associated microbial loads. The sensor system was also applied in a semi-industrial setting in a meat processing plant. Across replicate measurements, the assay provided consistent kinetic signatures and quantitative outputs, suitable for rapid and practical decision-making. Compared with traditional culture-based approaches (requiring up to 72 h), the respirometric sachets delivered actionable results within 10 h using a portable, low-infrastructure workflow, supporting rapid on-site hygiene verification and sanitation control.

## 1. Introduction

Bacteria can attach to abiotic surfaces and proliferate into structured communities known as biofilms, which are embedded in a self-produced extracellular matrix and exhibit enhanced tolerance to environmental stresses and antimicrobial treatments [1,2]. As a result, surface contamination by microorganisms represents a major challenge across healthcare, food production, and pharmaceutical industries [1,3,4]. Surface-associated microbial populations can reach densities of 10^4^–10^7^ CFU/cm^2^ under favourable conditions and are estimated to account for up to 80% of persistent contamination in industrial and clinical environments [2,5]. In healthcare settings, vegetative cells, bacterial spores, viruses, and yeasts can be readily transmitted from infected or colonized patients, healthcare personnel, and visitors, particularly via high-touch surfaces in close proximity to patients. Biofilm formation on medical devices and host tissues is a key factor in the onset of chronic and recurrent infections [4,5,6].

In the food sector, biofilms can adhere to a wide range of materials commonly used in food processing environments, including stainless steel and polymeric surfaces; contamination of food-contact surfaces is strongly influenced by biofilm development, which enables microbial persistence and survival under routine sanitation regimes [2,6], due to their intrinsic resistance to cleaning and disinfection procedures [1,3,5,6,7]. This persistence significantly increases the risk of cross-contamination and repeated contamination events, ultimately compromising food safety, shelf life, and regulatory compliance [6,7]. Importantly, biofilms may harbour foodborne pathogens such as *Listeria monocytogenes*, *Salmonella spp.*, and *Escherichia coli*, which are associated with severe foodborne illnesses, including listeriosis, salmonellosis, and enterohemorrhagic infections [2,5,8,9]. As a result, effective surface hygiene monitoring is critical for preventing contamination and ensuring food safety [5].

Surface hygiene control is a fundamental aspect of food processing environments that needs to be regularly monitored to satisfy the requirements set out in the Regulation (EC) No 852/2004 of the European Parliament and the Council on the hygiene of foodstuffs. Specifically, Annex II, Chapter II outlines the structural and hygienic requirements for food handling areas. Point (f) states that “surfaces (including surfaces of equipment) in areas where foods are handled and in particular those in contact with food are to be maintained in a sound condition and be easy to clean and, where necessary, to disinfect. This will require the use of smooth, washable corrosion-resistant and non-toxic materials, unless food business operators can satisfy the competent authority that other materials used are appropriate” [10]. This regulation underscores the critical role of microbiological monitoring of surfaces in ensuring food safety and compliance with Good Manufacturing Practices. The present study aligns with this framework by introducing a more rapid method for the assessment of bacterial contamination of surfaces than conventional culture-based methods.

Threshold values for acceptable microbial contamination on surfaces are not universally fixed and depend on the specific food sector and risk level [6,7]. The general requirements include among others the adequate maintenance of a clean condition of all rooms intended for food production, and food-contact surfaces must be easy to clean and disinfect by means of good hygienic operations. Food processing companies tend to establish technical specifications to add value to their products and limit contamination risks. Some studies have reported a quantitative aerobic colony count < 2.5 CFU/cm^2^ as post-cleaning microbiological surface standards for both the food and healthcare sectors [11,12]. However, different sectors such as fresh produce, dairy, beverage, and confectionery processing may adopt different criteria based on product characteristics, processing conditions, and regulatory or industry guidelines [13]. Sampling frequency and timing are typically defined within facility-specific hygiene monitoring plans (e.g., HACCP programs), rather than by the standard itself [14]. According to the guidelines of the Commission of the European Communities for hygiene in fresh meat processing facilities, food-contact surfaces exceeding this threshold are considered microbiologically unacceptable. However, these limits may vary depending on specific national regulations or industry standards [14,15].

ISO 18593:2018 is the primary reference standard for surface sampling in food processing environments, providing horizontal methods for sampling techniques in the food chain environment, with reference to any surface or item in contact with the food product or likely to represent a source of contamination. These methods are applied to detect and enumerate culturable microorganisms, including pathogenic and non-pathogenic bacteria, as well as yeasts and moulds [16].

The standard describes sampling procedures using contact plates, stick swabs, sponges, and cloths on a defined surface area (e.g., 100 cm^2^) following a systematic crosshatch pattern (horizontal, vertical, and diagonal strokes) to maximize microbial recovery. For larger or irregular surfaces, sponge-based sampling is recommended, involving thorough scrubbing followed by transfer of the sponge into a sterile container or bag for transport and analysis. After sampling, the sponge is transferred into a sterile bag containing neutralizing buffer or saline and homogenized in a flapper homogenizer to release the collected microorganisms, generating a suspension for subsequent analysis. This suspension is then subjected to standard microbiological testing according to established ISO methods for the enumeration of target microorganisms, typically involving serial dilution, plating on appropriate media, incubation under defined conditions for up to 72 h, and colony counting to obtain the total viable counts (TVC) on the swabbed surface. These reference procedures provide a quantitative benchmark for assessing surface hygiene and are widely used for the validation of alternative rapid detection methods [17,18]. However, traditional culture-based techniques [19,20,21], which rely on lengthy incubation times and require dedicated laboratory infrastructure, thereby limiting their suitability for rapid, on-site screening [22,23]. On-site screening offers significant advantages over centralized laboratory testing, including reduced time-to-result, improved process control through rapid decision-making, and enhanced ability to prevent cross-contamination via immediate intervention [24,25].

In response to the limitations of culture-based methods, several alternative approaches have been developed that exploit microbial metabolic activity as a proxy for viable cell load. These methods typically monitor biochemical processes associated with active metabolism, including oxygen consumption (respirometry), carbon dioxide evolution, ATP production, and redox transformations [26,27]. Among these, oxygen consumption represents an established operational parameter that quantifies the dissolved oxygen consumed by microorganisms during their growth in culture media (micro-respirometry, µR) [28]. Although primarily applied in water quality assessment, this principle highlights the potential of oxygen consumption as a rapid proxy for microbial activity, forming the basis for µR -based sensing approaches [18]. Sensor output can fall into a categorical contamination level (low/medium/high) where ‘high’ prompts further quantification with validated techniques [23].

Numerous oxygen-µR methods have been developed for microbial activity measurement in liquids, using electrochemical, optical, or pressure-based sensing (e.g., baroxymeters) [28,29]. However, these systems are typically designed for laboratory or wastewater applications, focus on bulk liquid samples, and often require trained personnel and multi-step workflows. To date, there is a lack of field-deployable, swab-to-µR platforms that can directly convert surface hygiene swabs into quantitative TVC results using a simple, disposable format. A wide range of plasmonic and other label-free optical biosensors (e.g., SPR, SERS, SEF, and related nano-plasmonic platforms) [18] have achieved remarkable sensitivity and, in some cases, very rapid detection of bacterial cells or their molecular signatures in well-controlled laboratory settings [30]. However, these approaches generally rely on sophisticated optical instrumentation, carefully engineered nanostructures, and specialized operators, which can limit their deployment for routine hygiene checks on food-contact surfaces, particularly in low-resource or small-scale settings.

The present work focuses on a non-specific µR platform that provides faster TVC estimates than the reference ISO method directly from surface swabs through the integration of a disposable sensor sachet and a portable optical reader designed for minimally trained users, making the platform suitable for on-site applications. The system is built from low-cost, commercially available components and utilizes standard sponge swab sampling in combination with sealable plastic sachets integrated with optical O_2_ sensors. The result is a compact, easy-to-use format for monitoring microbial oxygen consumption and estimating TVC in real time.

## 2. Materials and Methods

### 2.1. Preparation of Sensor Inserts

The sensor cocktail was prepared by dissolving 1.4 mg of Pt(II)-benzoporphyrin dye (Frontier Scientific, Newark, DE, USA) in 1.4 mL of a 5% (*w*/*w*) polystyrene solution (M.W. ~280,000, Sigma-Aldrich, St. Louis, MO, USA) in ethyl acetate. The mixture was sonicated for 15 min to ensure complete dissolution and homogeneity and subsequently dispensed in 3.0 µL aliquots onto sheets of microporous, low-fluorescence PVDF membrane (pore size 0.2 µm; Invitrogen, Milano, Italy), which were immersed in 70% (*v*/*v*) ethanol for 20 min, and then air-dried under sterile conditions. Sensor spots with a diameter of approximately 5 mm were thus produced, to match the geometry of the fibre optic port of the reader, ensuring reliable and reproducible optical alignment [31].

The coated membranes were air-dried for 30 min under a laminar flow hood, followed by thermal treatment at 70 °C for 3 h in a dry incubator, with samples protected by aluminum foil. The thermal treatment step was applied to ensure complete solvent removal, enhance polymer matrix stabilization and adhesion, and provide a mild pasteurization effect that minimizes potential microbial contamination of the sensor membrane prior to use [32,33]. The membranes were then stored in the dark until use.

Individual sensor patches (10 × 10 mm) were aseptically cut and affixed onto microporous medical-grade adhesive tape (3M Micropore™ 1530-1, Pioltello, Italy) to facilitate integration into the sensing platform. Sensors were evaluated for their phase shift (ΔΦ) and intensity (mV) signals under reduced partial pressure of O_2_ (kPa), following the recently published procedure for O_2_ barometric calibration [34].

### 2.2. Preparation of the Sensor Sachets

Mylar film sachets (85 × 140 mm external; 75 × 120 mm internal; 0.07 mm thickness; Wacomm Pack Inc., Kazo, Japan) with a clear front side, metallised back side, and resealable zipper lock were brought into a sterile biosafety cabinet. A single adhesive planar oxygen sensor sticker was aseptically affixed to the inner surface of each sachet to create single-use swab sachets for µR.

The sachets were intentionally designed with a non-selective formulation to capture the total aerobic microbial load, irrespective of taxa. These sachets function as a closed, diffusion-limited system with minimal headspace to enhance sensitivity to oxygen consumption.

**Figure 1:**
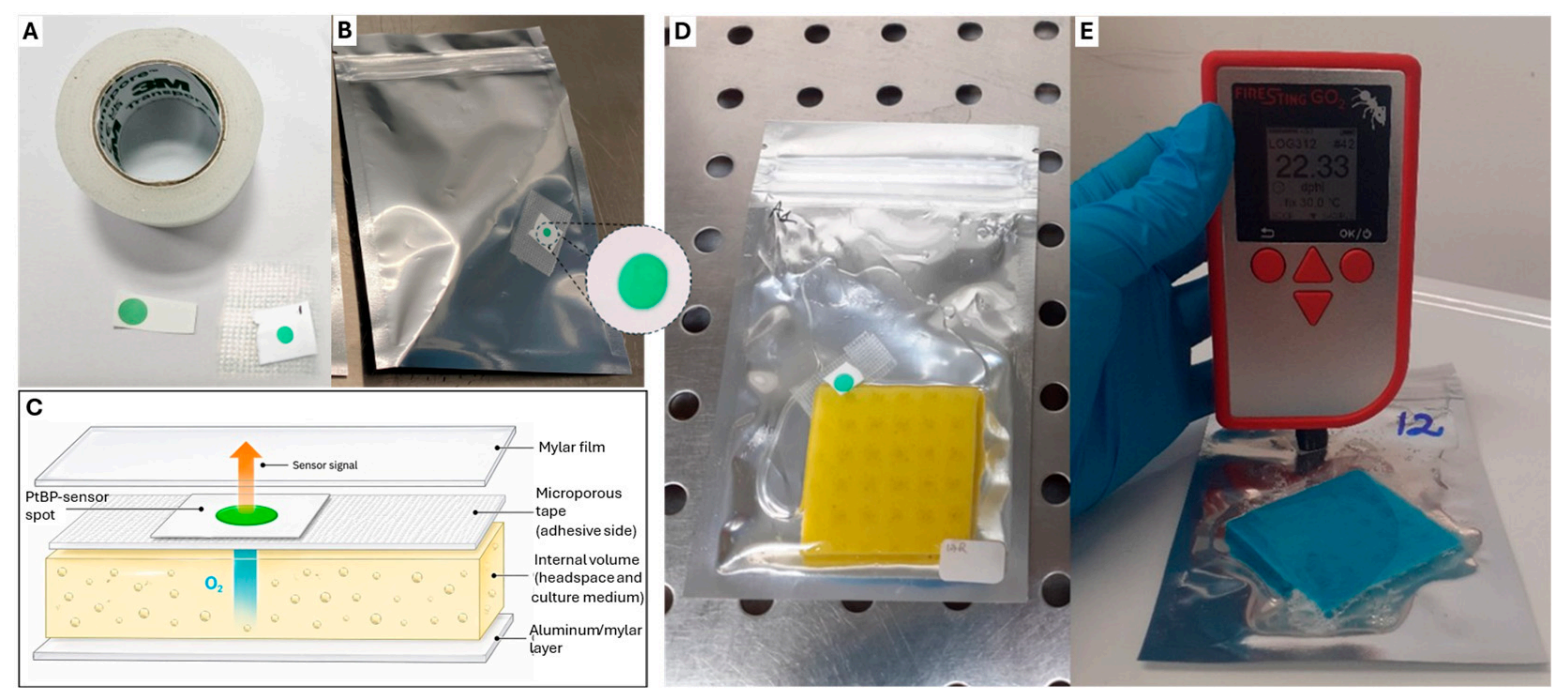
Components of the oxygen-sensing system for microbial detection on swabbed surfaces. (**A**) Assembly components, including microporous tape and sensor element prior to integration. (**B**) Sensor sachet incorporating the sensor sticker prior to culture media addition. (**C**) Schematic representation of the sensing configuration, showing the optical sensor positioned in the sachet and exposed to the internal headspace for monitoring O_2_ depletion during microbial respiration. (**D**) Final sensor sachet containing a sponge swab and culture medium, illustrating the complete swab-to-sachet setup used for microbial detection. (**E**) Non-destructive sensor signal measurements using the FSGO2 reader.

Batches of 20–30 sachets were quality assessed by measuring their intensity (mV) and phase shift (ΔΦ) signals with a FirestingO2 (FSGO2, Pyroscience, Aachen, Germany) handheld reader. The FireStingGO2 (FSGO2) employs phase fluorometry, measuring the phase shift (ΔΦ) of the luminescence signal, which is directly related to the sensor lifetime and enables measurements independent of intensity-related artefacts and sample heterogeneity. The device is handheld and autonomous, with internal data logging and display, and it is fully compatible with the PtBP-based sensing spots developed and calibrated in the laboratory, ensuring methodological consistency between benchtop and portable measurements [35,36]. All sensor readings were acquired non-invasively through the sachet wall (Figure 1E), without opening the sachet or removing the sensor, thereby preserving the sealed assay configuration throughout measurement. Each sensor measurement included three repeated readings with approximately 1 s intervals. The data logged into Firesting-GO2 Manager V1.1.1 (Pyroscience, Aachen, Germany) were then downloaded and analyzed in Excel.

The sterility of the sachets was assessed by aseptically dispensing 10 mL of Plate Count Broth (PCB; enzymatic digest of casein 5.0, yeast extract 2.5, glucose 1.0 g L^−1^, pH 7.0 at 25 °C) into each sensor sachet. The sachets were then sealed using a heat-sealing machine (Derikee Ltd., Manchester, UK) to ensure a secure and sterile closure and incubated at 30 °C for 72 h. After incubation, the sachets were examined for microbial growth and sensor signal variation, and those showing turbidity or sensor signal increase were considered contaminated and unsuitable for use. Unused sterile sachets were stored at 4 °C until use. Sponge swabs pre-moistened with saline (Microspec Ltd., Wirral, UK) were stored sealed in their original sterile packaging and opened immediately prior to sampling to preserve aseptic conditions.

### 2.3. Sensor Sachet Calibration with E. coli

*Escherichia coli* ATCC 8739 strain was used as a model aerobic microorganism to establish the sensor’s quantitative response (ΔΦ vs. log CFU/cm^2^), and to define the dynamic range and LOD_50_ under controlled conditions. This mirrors widespread practice in biosensor development, where a representative strain is used for calibration and proof-of-concept [22,37].

Ten mL of nutrient broth (NB, Oxoid/Thermo Fisher, Dublin, Ireland) was inoculated with a loopful of defrosted *E. coli* stock and incubated at 37 °C until an optical density (OD_600nm_) of 1.0 was reached (≈10^9^ CFU/mL). This suspension was used immediately to prepare a series of 1:10 dilutions in Buffered Peptone Water (BPW, enzymatic digest of casein 10.0, NaCl 5.0, Na_2_HPO_4_ 3.5, KH_2_PO_4_ 1.5 g L^−1^, final pH 7.0 ± 0.2 at 25 °C). One mL aliquots of each dilution were then dispensed in sterile sensor sachets containing the sponge swab and 9 mL of PCB. The sachets were then sealed, incubated at 30 °C, and measured hourly as described above. Time profiles of sensor optical signal (phase shift, ΔΦ) were produced for each sample, from which the time to reach the pre-set threshold of 25° ΔΦ [22,23] (TT) was determined. Thus, detection sensitivity is governed by metabolic activity rather than volumetric concentration effects. Each *E. coli* dilution was analyzed by µR in triplicate, while pure sterile PCB medium was used as a negative control. The TVC data produced with the sensor sachet method was benchmarked against ISO 4833-2 [19]. Preparation time for *E. coli* samples was standardized and kept to a minimum (5–10 min). Culture media, ingredients and BPW were from Biolife Italiana S.r.l. (Milano, Italy).

### 2.4. Surface Testing

The sensing assay was designed to mirror the growth conditions of the reference ISO method. Specifically, the same nutrient medium composition used for Plate Count Agar (PCA) in ISO 4833-2:2013 was employed in liquid form (i.e., without agar, PCB) within the sensor system. This approach ensures that the respirometric assay supports the same broad spectrum of aerobic microorganisms capable of growing under standard ISO-based culture conditions. Potential spore-forming bacteria commonly found on surfaces are not expected to substantially interfere with the measurements, since dormant spores are metabolically inactive and contribute to oxygen consumption only after germination. As no specific germination-inducing treatments are applied in the proposed assay conditions, the respirometric response primarily reflects the metabolically active microbial fraction present on the tested surface.

All surface swab samples were collected as specified in ISO 18593:2018 [16]. After disinfection with 96% ethanol, 22 × 10 cm test surfaces were prepared, divided with a masking tape into two 100 cm^2^ adjacent sections; this configuration was selected to standardize the sampled surface while increasing the total recovered biomass, thereby improving signal robustness and reproducibility. Each 10 × 10 cm area aligns with the surface units recommended in ISO 18593:2018, ensuring methodological consistency with established hygiene monitoring protocols.

The 220 cm^2^ area was then contaminated as follows: (i) by applying known concentrations of pure *E. coli* cultures directly onto the surface; (ii) by bringing fresh and spoiled raw meat in contact with the surface to introduce natural microflora typically found on meat-processing chopping boards; (iii) using raw fresh and spoiled vegetables as a source of environmental microflora typically found on vegetable-processing surfaces.

Artificial inoculation with *E. coli* was used to produce a wide range of contamination levels, which were subsequently quantified as TVC using both the ISO reference method and the sensor-based method.

When contaminating the surfaces with *E. coli* pure culture, 100 µL of bacterial suspension in BPW at a known concentration was evenly distributed across each 100 cm^2^ area using a sterile plate spreader.

**Figure 2:**
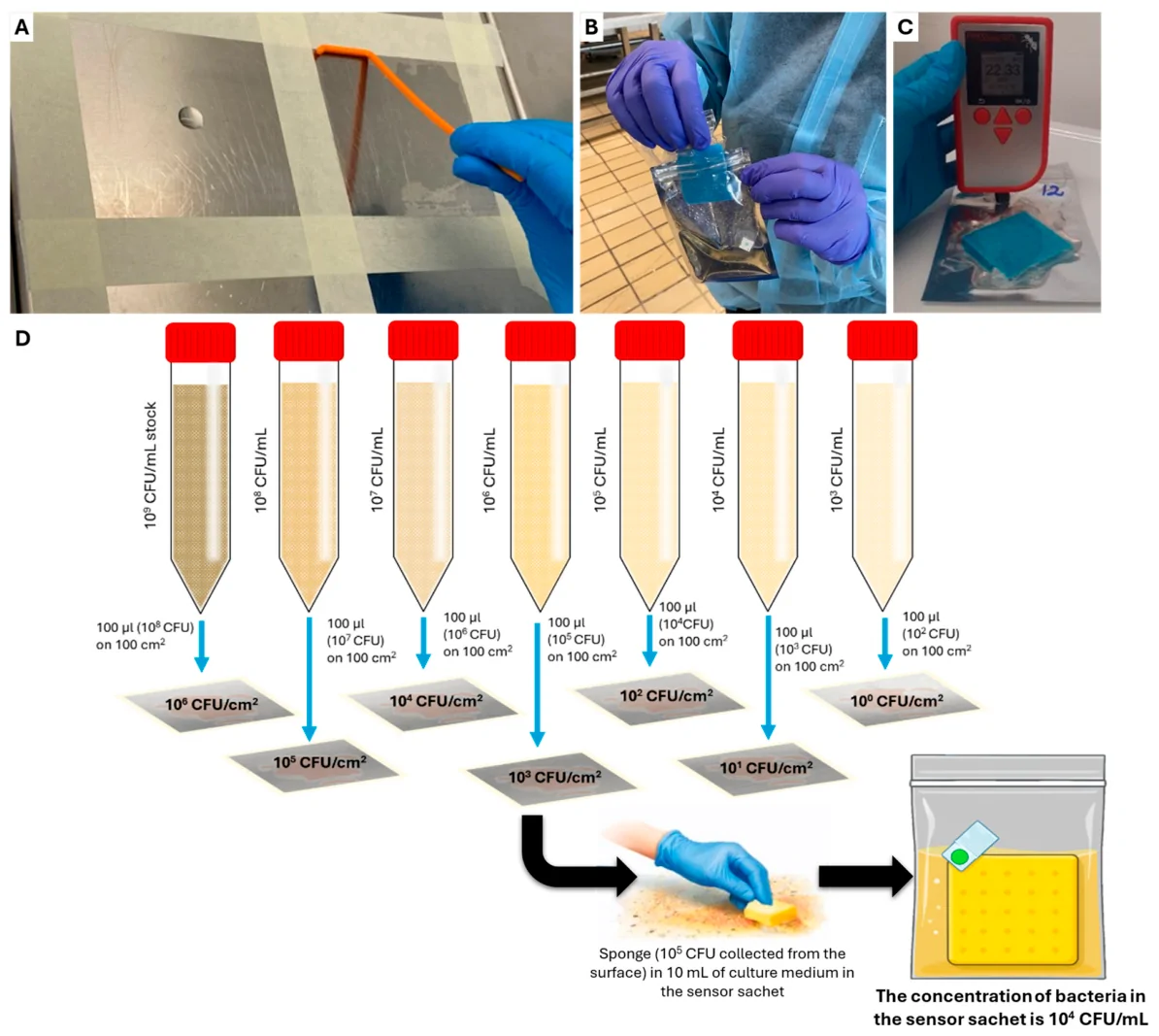
Test surface preparation, sensor sachets, and signal acquisition using the handheld reader. (**A**) Representative image of stainless-steel test surfaces, each marked as a 100 cm^2^ square. Surfaces were spot inoculated with 100 µL of either food extract or an *E. coli* suspension at varying concentrations for TVC assessment. Each area was processed using either ISO standard or the sensor-based respirometric method. (**B**) Insertion of the sponge swab into the sensor sachet containing pre-loaded culture medium and an oxygen sensor affixed to the inner Mylar surface. (**C**) Fully sealed Mylar/aluminum sachet prepared for incubation. Sensor spot (green) is visible through the transparent window. Real-time, non-destructive oxygen measurements are taken directly through the sachet using the FireStingGO2 optical reader. (**D**) Schematic showing the inoculation gradient of *E. coli* on test surfaces (10^7^ to 10^0^ CFU/cm^2^), simulating contamination scenarios for calibration and detection threshold determination. Samples were swabbed and transferred to sachets containing nutrient broth (NB) for microbial growth monitoring.

To simulate food-contact working surfaces (e.g., meat- and vegetable-processing lines), autochthonous microflora was transferred onto 22 × 10 cm stainless steel surfaces by repeatedly rubbing fresh pieces of meat or vegetables across the surface in four orthogonal directions, promoting a homogeneous microbial distribution and reproducing realistic contamination patterns encountered in food processing environments [22]. The inoculated surface was subsequently partitioned into two adjacent 10 × 10 cm sections using masking tape (Figure 2A). The paired areas were independently sampled and analyzed using the reference ISO method and the proposed sensor-based approach, respectively, enabling direct method comparison under identical contamination conditions. Sponge swabs were carefully removed from their sterile packaging and applied to each 10 × 10 cm area using a systematic back-and-forth motion (horizontal and vertical passes), in accordance with ISO 18593:2018. Each area was swabbed with multiple passes (*n* = 4) to maximize microbial recovery efficiency and ensure representative sampling of the defined surface [38].

For the ISO method, the swab sponges were transferred into homogenization bags containing 100 mL of BPW and processed in a flapping homogenizer (Stomacher) to release the collected microorganisms.

The resulting suspension was then subjected to serial dilutions in BPW and loading onto PCA plates by surface plating technique as specified in ISO 4833. After 48–72 h incubation at 30 °C, colonies were counted and bacterial load on test surface (TVC, CFU/cm^2^) was calculated according to the formula:

(1) $$\frac{CFU}{{cm}^{2}}=\frac{Average\;CFU/plate\;\times Volume\;of\;original\;suspension\times Dilution\;factor}{Swabbed\;surface\;area\;}$$

where

*Average CFU/plate* = Mean number of colonies counted from replicated plates at a given dilution;

*Volume of original suspension* = Total volume in which the swab was suspended (e.g., 100 mL of BPW);

*Swabbed surface area* = Typically 100 cm^2^ (10 cm × 10 cm), as per ISO 18593;

*Dilution factor* = The plated dilution (e.g., 10^1^, 10^2^, etc.)

The sensor sachet method follows a similar principle, with the workflow implemented in a single step using disposable sachets incorporating the O_2_ sensor for real-time and non-destructive monitoring of microbial respiration.

Each sensor sachet was pre-filled with 10 mL of PCB, as previously described [31]. Immediately after sampling, sponge swabs were transferred into the sachets, which were subsequently sealed. The sachet was then gently compressed using a coffee tamper to promote the release of microorganisms from the sponge into the medium.

Sterile sponge-in-sachet controls, prepared without surface sampling, were included as negative controls and processed under the same assay conditions.

Sensor readings were taken immediately after sealing the sachets, and then hourly during the incubation at 30 °C for up to 10 h. Batches of 10 sachets were read consecutively within 30 s, while maintaining them at 30 °C to minimize thermal fluctuations during the assay.

### 2.5. Application on a Meat Processing Plant

The swab-sachet sensor system was applied in the meat processing plant of Soalca S.r.l. (Pretoro, Italy). Surface sampling was performed by staff on equipment surfaces and operator interfaces, following internal standard hygiene monitoring procedures and in accordance with ISO 18593:2018. Sponge swabs were collected and immediately transferred into pre-filled sensor sachets containing culture medium, sealed, and incubated under controlled conditions. Oxygen consumption was monitored in situ using FSGO2 through phase fluorometric measurements (ΔΦ), and time-to-threshold (TT) values were determined based on the predefined signal threshold. As each sample was unique and not amenable to replication, 100 µL aliquots were taken from each sachet before incubation and used for analysis by the ISO reference method.

### 2.6. Data Analysis and Statistics

Sensor response curves obtained over a microbial load range of 0–7 log CFU/mL were constructed for each swab test, with three readings per hour averaged throughout the incubation period. These data were modelled using the four-parameter logistic function in DMFit v3.0 (ComBase, www.combase.cc), applying the Gompertz growth curve that models the sensor signal ΔΦ over time [33,39].

To define the optimal phase-shift threshold (ΔΦ) for calculation of the threshold time (TT), respiration profiles were analyzed at multiple candidate ΔΦ values. For each selected threshold, TT was defined as the time point corresponding to the onset of exponential oxygen depletion. The dependence of TT on threshold choice was then evaluated across all inoculum levels.

TT values were calibrated against both volumetric (logCFU/mL^−1^) and surface-normalized values (logCFU/cm^2^), with conversions accounting for sampled surface area and dilution factors. Calibration was based on *E. coli*, where TT values were correlated with corresponding TVC determined by the reference method on matched swabbed surfaces. Known *E. coli* concentrations (logCFU/cm^2^) were plotted against TT (h), and a linear regression model (Microsoft Excel) was used to derive the calibration equations, which were subsequently applied to estimate TVC in unknown samples. A polymicrobial calibration curve was established using native mixed microflora recovered from the tested surfaces, thereby accounting for community-level metabolic variability under realistic contamination conditions.

For quality control, readings from non-inoculated sensor sachets were analyzed to establish the in-control baseline. From these negative-control measurements, the mean (μ) and standard deviation (σ) were calculated. A signal threshold of 25° (>μ + 3σ) was then defined and used to determine the time-to-threshold (TT) from the respiration profiles [40,41].

The respiratory activity of the bacteria is expressed in terms of sensor signal as Φ(t) (the sensor signal at time t; then the TT (h) is:

$$\mathrm{TT}=t\;\mathrm{such}\;\mathrm{that}\;\Delta \Phi (t)\geq \;\Delta \Phi_{\mathrm{threshold}}$$

where:

ΔΦ(t) = sensor phase shift at time t (e.g., in degrees °)

$\Delta \Phi_{\mathrm{threshold}}$ = pre-defined threshold value for phase shift indicating microbial growth (e.g., 25°)

TT (defined in Section 2) showed an inverse relationship with microbial load. TT values can be interpolated between time points using curve fitting (e.g., 5-parameters logistic, Gompertz, etc.).

To assess the agreement between the sensor and conventional plating methods, Pearson correlation was used to assess the strength of the linear relationship between methods, while Deming regression was applied to evaluate agreement and calibration. Additionally, Bland–Altman analysis was performed to quantify bias and limits of agreement, to investigate on systematic differences and variability between the two methods [42,43,44,45]. Google Colab software was used (https://colab.research.google.com/, accessed on 7 April 2026)).

Unless otherwise specified, all values are reported as mean ± standard deviation of nine replicates (*n* = 9), obtained from three independent inoculation and sampling experiments conducted on different days. In contrast, samples collected from the meat processing plant were inherently unique and not amenable to replication.

## 3. Results and Discussion

### 3.1. Design of the Sensor Sachets and Measurement Set-Up for Swab Testing

This work introduces an integrated, field-adapted format consisting of an “all-in-one” swab-to-sachet workflow. The approach combines a disposable pouch embedding an optical O_2_ sensor with a handheld reader operating at a single ΔΦ threshold, enabling a one-step procedure with direct estimation of surface TVC and a time-to-result of <10 h. To our knowledge, this represents one of the first integrated swab-to-sachet platforms intended for on-site TVC estimation, combining ISO 18593-compatible swabbing with disposable optical respirometric sachets for more rapid TVC estimation.

Figure 1 presents a schematic of the sensor assembly. The O_2_-sensitive cocktail is dispensed onto a PVDF membrane to produce O_2_-sensing spot. The coated membrane is affixed to a medical-grade microporous adhesive tape and inserted within a sachet. The transparent front side of the sachet allows non-destructive optical measurements, while the metalized back side facilitates heat transfer and also enhances the intensity signal from the sensor. A sponge swab is also included. DF signals related to O_2_ levels are measured through the sachet using the FSGO2 phosphorescence detector, enabling non-invasive, real-time monitoring through the transparent window.

The sachet-based biosensor system utilizes adhesive planar optical O_2_ sensors [46] composed of PtBP embedded in an O_2_-permeable matrix (PVDF membrane and microporous tape) [34]. In the absence of oxygen, the PtBP dye exhibits strong phosphorescence when excited with modulated light. In the presence of oxygen, this phosphorescence is quenched via collisional (dynamic) quenching, which shortens the excited-state lifetime of the dye; Pt(II)-benzoporphyrin was selected because of its high oxygen sensitivity, long phosphorescence lifetime, excellent photostability, and suitability for phase fluorometric O_2_ sensing [47].

Oxygen concentration was measured using FSGO2 based on a two-point calibration (0 and 100% air saturation O_2_, corresponding to 0 and ~21% O_2_), according to the Stern–Volmer equation [48].

During microbial growth in the sealed sachet, oxygen is consumed by actively respiring bacteria, leading to a measurable decrease in O_2_ concentration over time. This O_2_ depletion curve serves as a surrogate for bacterial activity and is used to determine the threshold time (TT).

### 3.2. Quality Control Evaluation of Sensor Sachets and Sensor Calibration

QC testing of the sensor sachets (Supplemental Figure S1) demonstrated that the phase-shift signal (ΔΦ) remained tightly clustered over the 12 h monitoring period, with replicate-averaged values spanning ~19.97–20.63° and within-time point standard deviations of ~0.4–1.3°. Pooling all negative-control readings yielded an “in-control” baseline with mean μ ≈ 20.35° and σ ≈ 0.74°.

Sterility of the sealed sachet-based test units was evaluated by incubating uninoculated pouches containing sterile culture media and integrated oxygen sensors at 30 °C for 72 h. A total of 150 sachets were tested. Units displaying visible turbidity, gas formation, or sensor signals exceeding the baseline of 20.35 ± 0.74° ΔΦ were discarded. The remaining sachets showed no evidence of microbial growth or oxygen depletion and were subsequently stored at 4 °C until use. The stability of the baseline signal was also confirmed by daily measurements during the storage time (up to a month) [37].

Sensor drift and photobleaching effects were minimal over the assay timeframe, as indicated by stable blank responses. Overall, these factors did not affect the quantitative performance of the sensing platform. These findings validate the aseptic integrity of the sachet preparation process and refrigerated storage.

Negligible gas exchange through the polymeric barrier over the ≤4–6 h timeframe ensures that oxygen changes are primarily driven by microbial respiration, and not external diffusion. Overall, our findings validate the aseptic integrity of the sachet preparation process, even upon refrigerated storage [37].

### 3.3. Calibration Curve for TVC Quantitation in Swabs Using Sensor Sachets

Respiration profiles were obtained with *E. coli* standards to construct a calibration that allowed conversion of measured threshold time (TT, h) values into TVC (logCFU/cm^2^).

The experimental procedure for artificial surface inoculation of test surfaces and calibration is illustrated in Figure 2. Known quantities of *E. coli* were deposited on the test surfaces (10 × 10 cm, delimited by adhesive tape) as shown in Figure 2A. To validate the TVC sensor test in terms of accuracy, precision, and repeatability, we employed *E. coli* ATCC 8739—a reference strain commonly used for microbial enumeration and environmental hygiene monitoring. A concentrated cell suspension (~3 × 10^9^ CFU/mL), confirmed via plate counting on PCA, was prepared and serially diluted in tenfold steps. From each dilution, 100 µL were evenly distributed across 100 cm^2^ test surfaces to achieve surface loads ranging from ~1 to ~10^6^ CFU/cm^2^ (Figure 2B). Negative controls were performed by placing sterile sponges into the sachets without prior contact with any surface. These controls were processed in parallel with the surface swab samples and monitored under the same respirometric conditions. They did not produce a significant oxygen-consumption response, indicating that the observed signals in positive samples were attributable to microbial contamination collected from the tested surfaces rather than to the sponge or sachet components. Sensor signal was measured non-destructively every hour using a handheld FSGO2 reader (Figure 2C). Bacterial load on surfaces was calculated as shown in Figure 2D.

**Figure 3:**
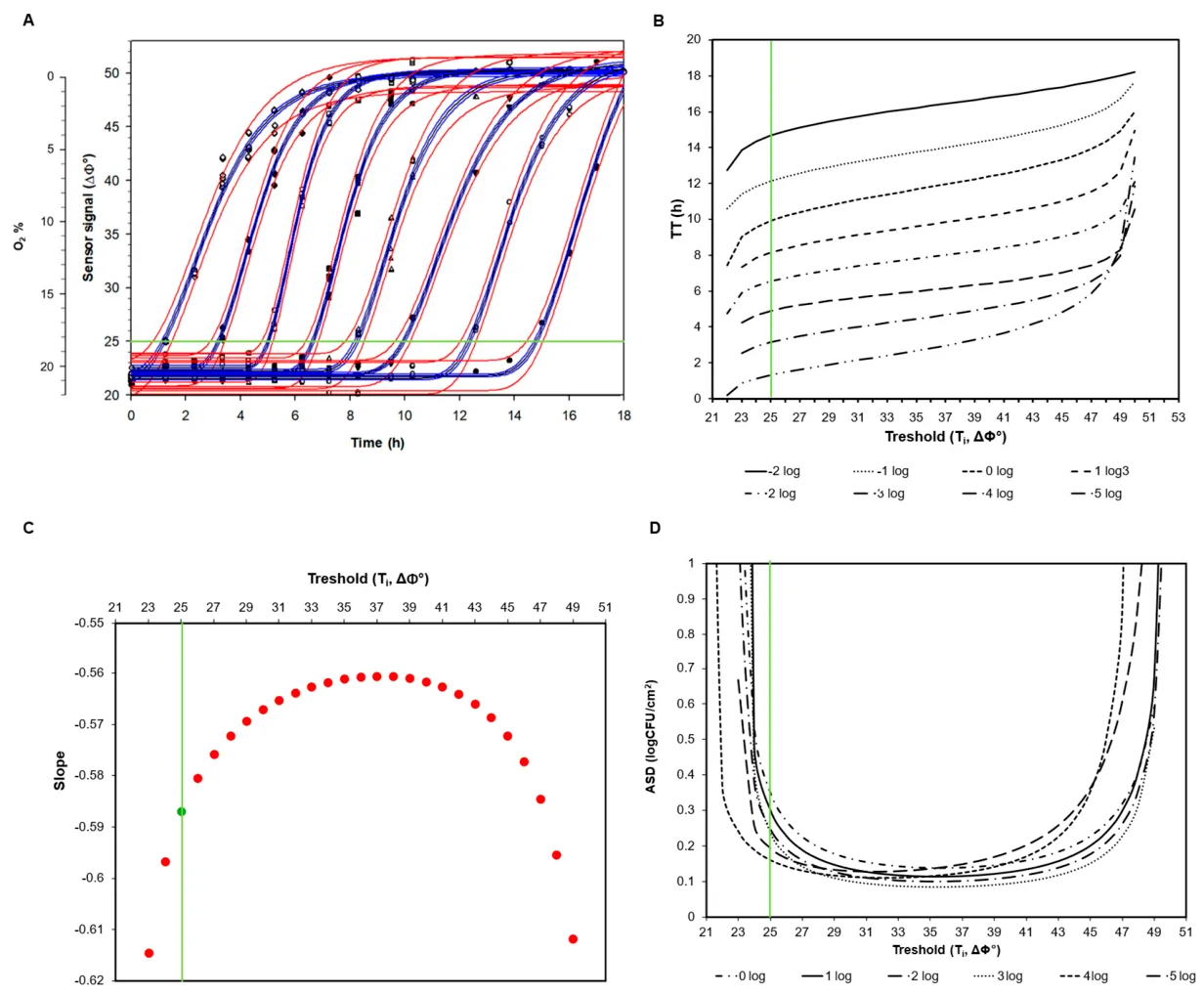
Respiration profiles of the sensor from calibration experiments and tested surfaces. The sensor response in the swab sachets inoculated with different known concentrations of *E. coli* ATCC 8739 is shown in (**A**) as sensor signal increase as ΔΦ(°) and as O_2_ depletion curves (pO2, kPa) for bacterial loads ranging from 0 to 7 logCFU/mL in the sensor sachets (corresponding to ≈ 1 CFU/cm^2^ to 6 logCFU/cm^2^ on the swabbed surface), from right to left, with higher concentrations leading to faster O_2_ consumption. Markers correspond to experimental data points. The fitted curves (black) follow the 4-parameter Gompertz model. The blue lines indicate the 95% confidence interval for the fitting, and the red lines represent the 95% prediction interval. (**B**) Dependence of TT on the selected ΔΦ threshold across microbial loads (0–7 logCFU/mL): low thresholds result in unstable TT values due to baseline effects, whereas increasing thresholds lead to a progressive increase in TT. (**C**) Dependence of calibration sensitivity on the selected phase threshold (ΔΦ): the slope of the TT–logCFU calibration curve is shown as a function of ΔΦ; the green dot indicates the slope at the chosen threshold of 25° (green line). (**D**) Dependence of quantification uncertainty on the selected phase threshold (ΔΦ°): the Average Successive Difference (ASD) of logCFU/cm^2^ estimates is reported for each threshold (Ti). A threshold of 25° (green line) was selected as an optimal compromise, providing stable and well-separated TT values while avoiding the delay associated with higher thresholds approaching signal saturation.

Respiration profiles at the different concentrations of *E. coli* in the sensor sachets are shown in Figure 3A. Sensor DF signals allowed tracing of residual O_2_ levels in tested samples. The sigmoidal curves indicate the respiratory activity of the bacteria, with increasing *E. coli* concentrations leading to a faster depletion of O_2_. As O_2_ concentration decreases from ~21% (air saturation) to ~0%, sensor phase signal increased from ~20 to ~51° degrees and intensity signal from 501 to 243 mV. The sensor response exhibited a clear saturation plateau at high TVC, corresponding to near-complete O_2_ depletion within the sealed sachet, over the dynamic range of 10^1^–10^6^ CFU/cm^2^ (Figure 3A).

As expected, lower bacterial loads in the sachets (e.g., 2 logCFU/mL) exhibit a slower response (longer TT values), whereas higher concentrations (e.g., 7 logCFU/mL) produce rapid sample deoxygenation (lower TT values).

The separation of the curves demonstrates the sensor’s ability to discriminate between different bacterial loads, confirming its suitability for indirect microbial enumeration.

A linear relationship between TT and TVC was observed, with shorter TT values corresponding to higher bacterial counts. The resulting equations enabled the transformation of sensor data into quantitative microbial estimates using the formulas (R^2^ = 0.99):

*TVC_sensor_* (*logCFU/mL*) = −0.51*TT*(*h*) + 7.03 (2)

*TVC_sensor_* (*logCFU/cm*^2^) = −0.51*TT*(*h*) + 6.03 (3)

The CFU/mL Equation (2) refers to the microbial concentration within the sachet assay medium, whereas the CFU/cm^2^ Equation (3) refers to the corresponding microbial load recovered from the sampled surface and normalized to the sampled area. The difference in the intercepts reflects the conversion from the microbial concentration measured in the sachet medium to the microbial load normalized to the sampled surface area. Since a 100 cm^2^ surface was sampled and the sponge was placed into 10 mL of medium, the corresponding surface load is calculated as CFU/cm^2^ = (CFU/mL × 10 mL)/100 cm^2^, i.e., CFU/cm^2^ = 0.1 × CFU/mL. On a log10 scale, this conversion corresponds to a decrease of 1 log unit. Therefore, the intercept of the CFU/cm^2^ equation is 1 log lower than that of the CFU/mL equation, while the slope remains unchanged because the same respirometric threshold-time response is being described.

The dependence of TT on the selected ΔΦ threshold was evaluated across a range of microbial loads (Figure 3B). At low threshold values (≤23°), TT exhibited abrupt transitions and limited separation between curves, indicating greater influence of baseline noise and less reliable TT determination. In contrast, intermediate thresholds (~23–29°) produced smooth and well-separated TT profiles, with similar slope values across contamination levels. At higher thresholds (>30°), TT increased progressively for all curves, resulting in delayed detection and partial convergence between microbial loads, consistent with the signal approaching its saturation region.

The relationship between quantification error (ΔlogCFU/cm^2^) and calibration sensitivity (slope) as a function of the selected phase threshold (ΔΦ) reveals a clear trade-off (Figure 3C). While minimum error and maximum slope are achieved at higher thresholds (≈33–38°), these conditions correspond to longer detection times. In contrast, lower thresholds (<24°) are affected by baseline instability. The intermediate region (25–30°) defines an optimal domain, where improvements in accuracy are achieved with minimal penalty in assay duration. Notably, ΔΦ = 25° represents the point beyond which further gains in accuracy become marginal relative to the increase in response time.

Based on these observations, a threshold of 25° was selected as an optimal compromise, ensuring a high signal-to-noise ratio and robust discrimination from baseline fluctuations. The chosen threshold value was further corroborated by visual inspection of respiration profiles from inoculated samples, where the 25° ΔΦ consistently coincided with the onset of measurable oxygen depletion associated with microbial activity: a ΔΦ of 25° corresponds to approximately 18% O_2_, within the dynamic region of the sensor response where O_2_ depletion is clearly detectable before sensor saturation.

In the context of Figure 3D, the Average Successive Difference (ASD) quantifies the consistency of change between successive threshold-time points in each curve [49]. It reflects the system’s sensitivity to microbial activity over time and helps differentiate between contamination levels. The ASD is calculated as:

(4) $$ASD=\frac{1}{n-1}\sum_{i=1}^{n-1}\left({TT}_{i}+1-TT_{i}\right)$$

where *TT_i_* = the value at time point *i*; *n* = total number of time points; and T*_i+_*_1_ − T*_i_* = difference between successive measurements.

The sum of these differences is divided by *n* − 1, the number of intervals between *n* observations.

The ASD between *E. coli* respiration curves is 1.94 ± 0.35 h, meaning that for each log increase in initial TVC, the time required for the sensor to detect TT shifts is approximately 2 h. This consistent interval highlights the system sensitivity and ability to discriminate between contamination levels from the sensor signal (Figure 3B). This also represents a significant reduction in detection time compared to ISO method, enabling near real-time assessment of highly contaminated surfaces. Furthermore, the system shows high temporal resolution and predictable growth-linked signal delays, ideal for modelling or thresholding detection windows.

### 3.4. Analysis of Surfaces

**Figure 4:**
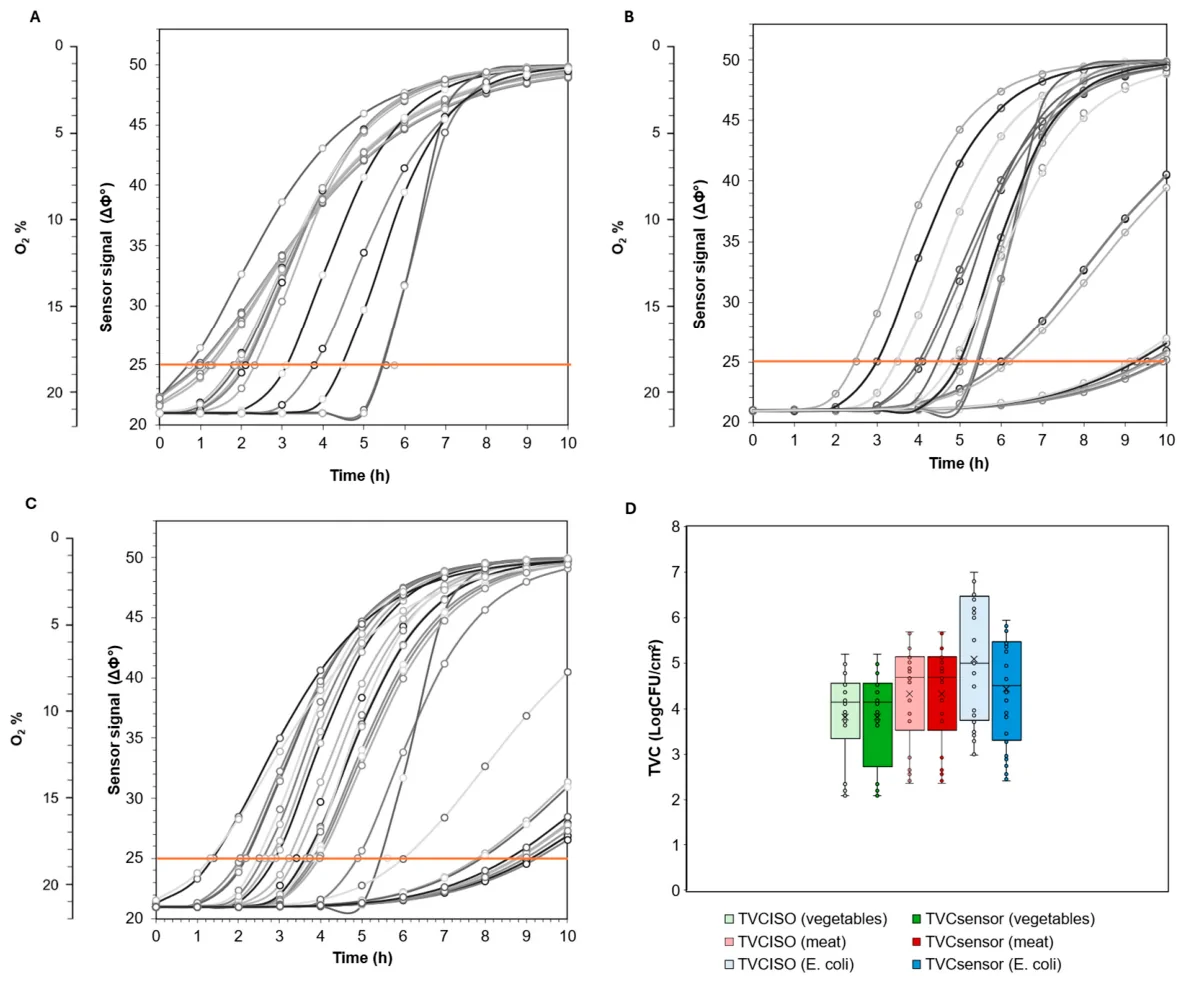
Respiration profiles of the sensor from tested surfaces. (**A**) Respiration profiles from surfaces artificially contaminated with different concentrations of *E. coli*. (**B**) Respiration profiles from surfaces artificially contaminated with vegetables. (**C**) Respiration profiles from surfaces artificially contaminated with meat. The threshold lines at 25° are shown in orange. (**D**) Box plot comparing total viable count (TVC) (logCFU/cm^2^) between two methods from the analysis of 90 different test surfaces artificially inoculated with *E. coli* cells, food extracts from meat and vegetable origins. The horizontal lines within the boxes represent medians, the × represents average, the box width represent the interquartile range, the whiskers extend from the lower to upper range. Outliers appear as individual dots beyond the whiskers.

The validation of the method was carried out by analyzing artificially contaminated surfaces. Figure 4A–C shows respiration profiles from surfaces artificially inoculated with *E. coli*, vegetables and meat microflora. These curves were used to obtain TT values to quantify TVC in the tested surfaces using the calibration Equation (2) or (3).

The respiration curves followed the four-parameter model as described in previous studies [22]. In most cases (especially from TVC_ISO_ 3.0 to 5.0 log CFU/cm^2^), the sensor estimates were within ±0.3 log units, which is considered acceptable in microbiological quantification. This indicates that the sensor performed most reliably in the mid-log range, which corresponds to typical surface contamination in food or environmental hygiene monitoring.

The box plots in Figure 4D compare the TVCs of bacteria on vegetable- and meat-treated surfaces measured by ISO 4833-2 (TVC_ISO_) and the sensor-based method (TVC_sensor_), both sampled according to the ISO 18593 method. Oxygen consumption profiles reflect microbial respiration under the defined assay conditions. In fact, both methods show a similar range of TVC values, with overlapping interquartile ranges (IQRs), suggesting good agreement. The median values (central horizontal line within the boxes) are nearly identical for each food type across both methods. The higher median TVC on meat-microflora-treated surfaces (4.61 logCFU/cm^2^ for TVC_sensor_ and 4.35 logCFU/cm^2^ for TVC_ISO_, red box plots) than on vegetable-treated surfaces (3.92 logCFU/cm^2^ for TVC_sensor_ and 4.00 logCFU/cm^2^ for TVC_ISO_, green box plots) and surfaces contaminated with *E. coli* (4.38 logCFU/cm^2^ for TVC_sensor_ and 4.00 logCFU/cm^2^ for TVC_ISO_, blue box plots) suggests that meat-treated surfaces tend to harbour more bacteria.

The spread of data in the TVC_sensor_ method appeared slightly larger in some cases, which may indicate minor variations in sensor performance or differences in sample collection efficiency. The presence of outliers (small circles outside the whiskers) indicates variability in contamination levels, possibly due to varying initial contamination or uneven bacterial distribution on surfaces.

As generally expected for the swabbing procedure [38], after sampling the same surface spot 4 times, approximately 75–90% of the microorganisms were recovered. Details on the inoculum types on the tested surfaces are reported in Supplemental Table S1.

While the potential for matrix effects—such as interference from residual animal or plant cells affecting oxygen diffusion or microbial growth—was considered, as also shown in our previous studies [22,23,32,50], no systematic impact on the sensor’s performance was observed within the tested matrices. Across diverse sample types, including raw meat, leafy vegetables, and food-contact surfaces with visible organic residues, the TT values remained correlated with TVC, with no systematic deviation or signal suppression attributable to matrix composition. These findings support the robustness of the sensor sachet method in real-world hygiene monitoring applications, even in the presence of complex surface residues.

In polymicrobial surface samples, we observed occasional downward trends or signal fluctuations in the oxygen profiles beyond the exponential phase, which were not present in tests with pure *E. coli* cultures (Figure 3). This phenomenon is likely attributable to the presence of slow-growing or metabolically distinct aerobic species that initiate respiration at later time points. Oxygen uptake rates and specific rates of oxygen consumption vary between bacterial species and growth states, with some bacteria (e.g., *Pseudomonas* species) consuming oxygen more rapidly than others under similar conditions [37]. Such variability indicates that the respirometric signal does not represent a fixed per-CFU parameter but rather an emergent property of the community’s metabolic activity over time [51]. In this context, the use of a polymicrobial calibration curve accommodates the composite respiration behaviour of complex surface communities more effectively than a single-strain calibration alone.

### 3.5. Comparative Analysis of the Sensor-Based and ISO Standard Methods

**Figure 5:**
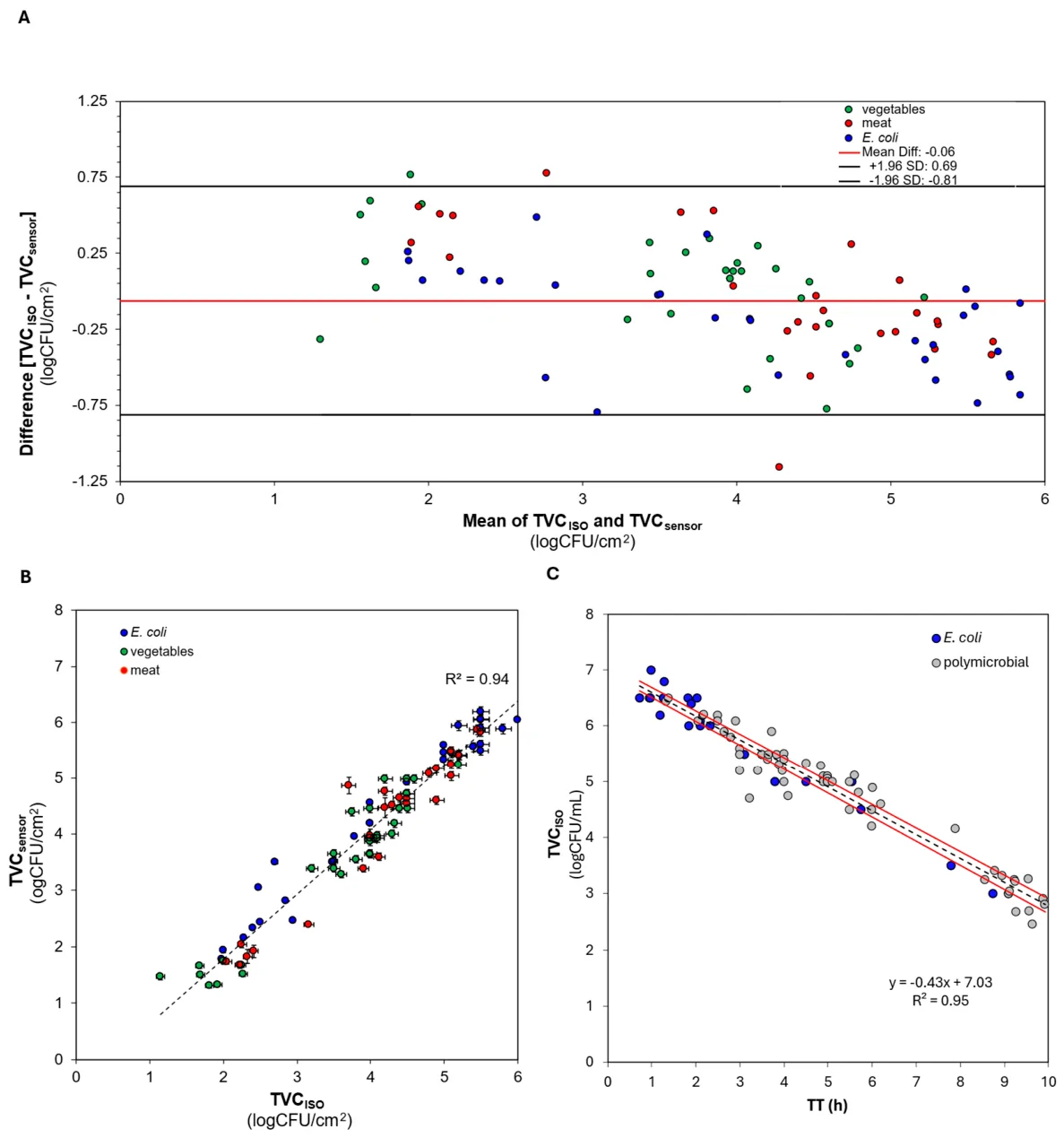
Comparison of TVC_ISO_ and TVC_sensor_ methods using Bland–Altman and Pearson correlation analysis. (**A**) Bland–Altman plot assessing the agreement between the ISO 4833-2 reference method and the swab-sachet sensor method. The mean difference (red line) is close to zero, with 95% limits of agreement (black lines) indicating the range within which most differences fall. (**B**) Pearson correlation plot of data comparing data from vegetables and meat contact surfaces, with values from both methods; error bars represent standard deviations. (**C**) Calibration derived from combined dataset (*n* = 90 samples) plotting threshold time (TT, hours) data with the relevant counts obtained from the ISO method for *E. coli* and polymicrobial samples from each tested surface (vegetables and meat contact surfaces). Dashed line represents calibration curve, and the red lines represent the 95% confidence limits.

Statistical correlation analysis was performed to evaluate the agreement between the TVC_sensor_ method and the TVC_ISO_ reference method. The results in Figure 5 indicate a strong positive correlation between the two approaches, demonstrating that the TVC_sensor_ method effectively replicates the bacterial enumeration trends observed using the standard TVC_ISO_ method.

Bland–Altman analysis was used to assess agreement between the sensor method and the ISO 4833-2 reference method by evaluating the bias and limits of agreement (LoA) between paired measurements (Figure 5A).

The mean difference (bias) between the sensor and the ISO method was +0.06 log CFU/cm^2^, indicating a slight average overestimation by the sensor. The 95% LoA ranged from −0.69 to +0.81 logCFU cm^−2^, with 97% of data points falling within this interval, demonstrating good agreement between the two methods. These differences are consistent with the intrinsic variability of culture-based microbiological enumeration, which is influenced by sampling efficiency, plating variability, and biological heterogeneity [19,20,52]. The Bland–Altman plot shows that the differences between methods are randomly scattered across the range of mean values, with no clear trend, thus supporting the absence of proportional bias. This implies that the agreement between methods remains stable across varying microbial concentrations. However, a wider spread of differences is noticeable at higher mean values, suggesting increased variability in sensor readings at elevated contamination levels. While no consistent trend in bias is evident across different sample types (Mean Difference = −0.06). The systematic bias—where the sensor method reports slightly higher counts than the ISO method—may reflect its ability to detect stressed cells in the liquid media that the plate count involved in the ISO method misses. This difference is an inherent characteristic of the two methodologies and should be considered in applications where the full spectrum of viable cells is of interest. In environments like food production or clinical settings, where quick decisions are crucial, having a validated on-site compatible method that correlates well with the ISO standard can enable more frequent monitoring, early detection of contamination issues, and faster corrective actions.

Pearson correlation and Deming regression were used to assess the relationship between the sensing platform and conventional plating results. Pearson correlation quantifies the strength of the linear association between the two methods, while Deming regression accounts for measurement errors in both variables, providing a more appropriate estimation of agreement when both methods are subject to uncertainty [53]. Together, these complementary approaches enable a comprehensive evaluation of correlation, accuracy, and interchangeability between the sensor-based measurements and standard plate counts [43,44,54]. The Pearson correlation analysis is shown in Figure 5B. The scatter distribution of TVC values from both methods suggests a very strong linear relationship (r = 0.97, *p* < 0.01). However, slight discrepancies were observed at higher TVC values, where the sensor method exhibited marginally greater variability. The regression line shows a positive intercept for the dataset of 0.53 with a slope of 1.15, indicating that the sensor method consistently reports slightly higher values than the ISO method, regardless of the actual measurement level. This offset may be due to the fact that stressed bacteria, which reportedly might not grow well on solid agar media [55], can still be metabolically active and detected in liquid media in the sensor sachets. Despite these minor differences, the high correlation coefficient reinforces the findings of the Bland–Altman analysis and confirms that the sensor-based method a promising complementary approach to the ISO method, offering significant advantages in real-time bacterial surface monitoring.

The regression plot in Figure 5C shows TVC vs TT (h) for both groups, with separate regression lines and 95% confidence intervals. The visual confirms the statistical findings, showing overlapping trends and similar curve shapes.

### 3.6. Polymicrobial Calibration Curve for TVC Quantitation

To compare the TVC values obtained by the ISO plate-counting method with those estimated by the TVC sensor-based method, 90 stainless-steel surface samples were artificially contaminated by rubbing meat and vegetable samples. In parallel, ethanol-disinfected clean stainless-steel surfaces were inoculated with known concentrations of *E. coli* ATCC 8739. Since both the food-contaminated and *E. coli*-inoculated surfaces were divided into two equal areas, one half could be analyzed by the ISO plate-counting method and the other by the sensor-based method. This experimental design allowed us to assess whether a calibration curve generated using the representative microorganism *E. coli* ATCC 8739 could accurately estimate TVC in samples containing heterogeneous, naturally occurring food-associated microbial communities. Figure 5C shows the calibration curve generated from data obtained from 90 food-contaminated surface samples and surfaces artificially inoculated with *E. coli*, plotting threshold times (TT, h) versus TVCs obtained according to the ISO method. The new polymicrobial calibration equations (R^2^ = 0.95) for surface testing with the sensor swab-sachet method are:

*TVC_sensor_* (*logCFU/mL*) = −0.43*TT*(*h*) + 7.04 (5)

*TVC_sensor_* (*logCFU/cm*^2^) = −0.43*TT*(*h*) + 6.03 (6)

The use of a naturally contaminated polymicrobial sample set for calibration was intended to incorporate part of the biological variability associated with mixed aerobic microbiota, rather than relying exclusively on a single-species respiratory profile.

To determine whether calibration curves derived from TT measurements can be used interchangeably for *E. coli* and polymicrobial samples when interpolating TVC, an Analysis of Covariance (ANCOVA) was conducted. TT was used as the independent predictor variable and TVC as the dependent variable in the ANCOVA model; the resulting regression was subsequently used to estimate TVC from measured TT values (Table 1). The grouping variable distinguished between *E. coli* and polymicrobial samples. An interaction term (TT × Group) was included to test for slope differences. ANCOVA assumptions were confirmed by residual diagnostics, with no evidence of heteroscedasticity and only minor deviations from normality (including normality of residuals, homoscedasticity, and independence); supporting plots are shown in Supplementary Figure S2. The analysis showed that TT is a highly significant predictor of TVC, confirming its utility in microbial quantification. The group effect was not significant (*p* = 0.658), indicating no meaningful difference in intercepts between *E. coli* and polymicrobial samples. Finally, the interaction term was not significant (*p* = 0.256), suggesting that the slope of the calibration curve does not differ significantly between groups.

Overall, the ANCOVA results support the comparability of calibration curves for *E. coli* and polymicrobial samples within the tested conditions. Both curves share similar slopes and intercepts, validating the use of a unified calibration model for interpolating TVC across these sample types.

These findings are supported by previous experimental studies combining respirometric measurements with microbial identification techniques (e.g., 16S rRNA gene sequencing) [56], demonstrating that oxygen consumption capture the global metabolic activity of complex microbial communities in the tested samples, and correlate with total bacterial load. Thus, µR reflects the collective metabolic activity of the microbial community, rather than the behaviour of any individual dominant species [56].

Logistic regression models [57,58,59] were applied to the detection data from both the reference and sensor methods, using the presence or absence of a microbial signal (above a 2.5 logCFU/cm^2^ threshold) as a binary outcome using a binomial logit link function (Table 2). For each method, the regression output yielded an intercept (α) and slope (β), which were then used to calculate the LOD_50_ (the contamination level corresponding to a 50% detection probability). The relative limit of detection (RLOD) was computed as the ratio of LOD_50_ for the TVC_sensor_ method to that of the TVC_ISO_ reference.

Results showed an LOD_50_ of 2.69 logCFU/cm^2^ for the TVC_sensor_ method and 2.45 logCFU/cm^2^ for the TVC_ISO_ method. The relative limit of detection (RLOD = 1.10) indicated a marginal increase (~10%) in the detection limit of the sensor method compared to the ISO method, consistent with the Bland–Altman analysis, which revealed minimal bias (+0.06 logCFU/cm^2^) and narrow limits of agreement (−0.69 to +0.81 logCFU/cm^2^). These results fall within the expected variability of culture-based microbiological assays, supporting acceptable equivalence between methods [52].

In terms of diagnostic performance, the intrinsic sensitivity of the sensor-based method was 0.93, reflecting its ability to detect microbial presence based on the defined threshold criteria, with 93% of positive samples correctly identified. When evaluated relative to the ISO method, the sensitivity reached 1.00, indicating that all samples classified as positive by the ISO method were correctly detected by the biosensor (i.e., no false negatives relative to the reference). The Relative Trueness (RT) represents the proportion of results from the sensor method that match those obtained with the reference method (ISO), across the tested range. We obtained a value of 0.94, thus demonstrating strong agreement between the two methods across the tested range, reinforcing the sensor’s validity for quantitative applications, especially for field-deployable diagnostics, indicating that the sensor method produces results that are reliably comparable to standard lab procedures.

The false positive rate (FPR) refers to the proportion of negative samples (according to the ISO method) that were incorrectly identified as positive by the sensor-based method. None of the samples that were negative according to the ISO 4833:2013 [19,20] method was incorrectly flagged as positive by the sensor sachet system (FPR_sensor_ = 0.00). A zero false positive rate is highly desirable, especially in hygiene monitoring and food safety, where false positives can lead to unnecessary product rejections, costly recalls, or over-sanitation.

The Mean Absolute Error (MAE) was 0.31 log CFU/cm^2^, and the Root Mean Squared Error (RMSE) was 0.38 log CFU/cm^2^, indicating a generally low level of deviation between the two methods. The Mean Relative Error (MRE) was −0.33%, suggesting no significant systematic over- or underestimation. Furthermore, 98.89% of the sensor readings fell within ±1 log unit; this corresponds to a tenfold difference in bacterial counts (0.1×–10× of the reference value), which is widely accepted in microbiological analyses given the intrinsic variability of culture-based methods [12,52]. The observed analytical variability (RMSE = 0.38 log CFU/cm^2^) is consistent with expected inter-method variability in microbiological enumeration; the sensor method also showed good repeatability, with an RSD below 5% for all tested samples [52].

Overall, the sensor-based method demonstrated high analytical performance and agreement with the reference ISO method, reliably discriminating between contaminated and clean surfaces without generating false-positive results. The analytical selectivity of the proposed biosensor is a critical requirement for real-time, on-site microbial screening and supports the use of the biosensor as a promising platform for a more rapid on-site estimation of TVC, showing good agreement with the ISO reference method while offering portability, operational simplicity, and reduced time-to-result, as well as the additional advantages of portability, operational simplicity, and significantly reduced time-to-result.

Furthermore, while the current implementation targets total aerobic viable counts, the platform’s modular design opens the possibility for future adaptation with selective media or receptor-based elements to achieve pathogen-specific detection.

### 3.7. Operational Performance and Scalability

The use of a swab-sachet sensor system during this pilot field evaluation in the meat-processing environment allowed for a rapid estimation of the level of surface contamination in both post-cleaning and use conditions (Table 3). Immediately after cleaning, surfaces (swabs 1B, 4B and 6B) showed no detectable contamination (19 h). On the other hand, moderate contamination levels were found on the surfaces of the preparation room (Swabs 2B and 3B) with TT values of 11.65 and 10.91 h, corresponding to 0.09 and 0.47 logCFU/cm^2^, respectively. Substantially higher contamination levels were observed during processing on critical contact surfaces, including knives and operator gloves (swabs 7B and 8B) with very low TT values (0.55 and 0.22 h) and correspondingly high estimated load values (5.75 and 5.92 logCFU/cm^2^). These results highlight the ability of the system to distinguish between low, medium and high contamination scenarios and to rapidly identify critical points of risk in the processing process.

To evaluate the performance and practical benefits of the proposed O_2_ µR-based sensor sachet method, a detailed, time-resolved comparison was conducted with the conventional ISO 4833-2 reference method for microbial enumeration on food-contact surfaces. Table 4 outlines the complete workflow for each method, from surface preparation and swabbing to microbial analysis and time-to-result. While both approaches share initial steps such as surface inoculation and swab sampling, the sensor sachet method introduces a simplified, single-step analytical format that eliminates the need for homogenization, serial dilution, agar plating, and prolonged incubation.

While the ISO method quantifies TVC (in CFU/mL or CFU/cm^2^) through manual colony counting (see Equation (1)), the sensor sachet method applies calibration curves to convert time-to-threshold (TT) values—representing the point of detectable O_2_ depletion—into equivalent TVC values. For this reason, calibration curves must be drawn carefully and periodically verified.

Overall, the sensor-sachet approach substantially reduces both hands-on time and total analysis duration, allowing microbial quantification within hours compared to the 48–72 h required by the standard method. In addition to reduced complexity, the sensor-based approach demonstrates strong potential for automation and scalability. A cost analysis is shown in Supplementary Table S2 and indicates an estimated cost of ~6.5–7.5 € per sample compared to ~8–16 € for culture-based assays, while also reducing labour requirements. The system is inherently scalable, as multiple sachets can be processed in parallel without specialized infrastructure, enabling high sample throughput and supporting large-scale or field-based applications.

Furthermore, the simplicity of the workflow and the non-invasive optical readout facilitate integration with automated and digital monitoring systems for remote or online monitoring and control of surface contamination. The time-resolved signals (e.g., TT and oxygen consumption profiles) can be readily interfaced with data acquisition platforms and are amenable to machine learning-based analysis for predictive modelling and anomaly detection. Combined with its portability and minimal infrastructure requirements, the platform is well-suited for remote or on-site deployment, with potential integration into connected monitoring frameworks for real-time or near real-time assessment of surface contamination.

## 4. Conclusions

This study evaluates a µR-based sensor sachet platform for facile estimation of total viable counts (TVC) on environmental surfaces. The method estimates TVC indirectly via oxygen consumption. Quantitative performance was established through calibration against the ISO 4833:2013 reference method, showing strong correlation (R^2^ > 0.94) and agreement (bias = +0.06 logCFU/cm^2^; limits of agreement −0.69 to +0.81 log CFU/cm^2^) across the tested range (~10^1^–10^6^ CFU/cm^2^). The relative limit of detection (RLOD = 1.10) and reproducibility across independent experiments further support the analytical consistency of the approach under the defined conditions. Within its current scope, the method is best positioned as a complementary approach to conventional culture-based methods, providing rapid estimates of total aerobic TVC reducing analysis time (≤10 h versus 24–72 h for culture-based methods), simplified handling, and competitive cost. However, further validation is required to assess robustness across different surfaces, environmental conditions, and independent laboratories to support routine deployment.

Because oxygen-consumption rates may vary among microbial taxa and physiological states, the calibration equation should be considered matrix- and condition-specific, providing a TVC-equivalent estimate rather than a universal conversion of respiration into viable cell numbers.

Detection is currently limited to aerobic microorganisms, and signal dynamics may be influenced by matrix effects such as surface residues or complex sample composition. In addition, the method’s limit of detection (~2.7 logCFU/cm^2^) may restrict its applicability for ultra-clean environments requiring detection below this threshold.

From an operational perspective, the platform offers reduced analysis time (hours versus 24–72 h for culture-based methods), simplified handling, and competitive cost (~6.5–7.5 € per sample). The ability to process multiple sachets in parallel supports scalability and increased sample throughput. However, further validation is required to assess robustness across different surfaces, environmental conditions, and independent laboratories. Additional studies addressing sensor stability, shelf life, and performance under variable temperature conditions will be necessary to support routine deployment.

Nevertheless, broader adoption will require systematic inter-laboratory validation and benchmarking against established reference methods to demonstrate reproducibility, robustness, and fitness for purpose under real-world conditions. Future validation in accordance with ISO 16140 [57] frameworks will be essential to support regulatory acceptance.

Overall, the proposed platform represents a promising and practical tool for rapid screening of surface contamination. The results obtained at the meat processing plant show that the swab-based sensor system is capable of rapidly detecting and distinguishing the levels of contamination on different surfaces, allowing for the early identification of critical risk points in the processing environment. Within its current scope, the method is best positioned as a complementary approach to conventional culture-based methods, providing more rapid estimates of total aerobic TVC than the ISO method, and facilitating earlier identification and monitoring of contamination risks, while further validation and standardization efforts are pursued. Future studies may investigate the application of the proposed biosensor in combination with molecular methods (e.g., 16S rRNA sequencing) to characterize microbial community composition.

## Figures and Tables

**Table 1:** ANCOVA comparing calibration curves for *E. coli* and polymicrobial samples. TT = time to threshold (h); TVC = total viable count (log CFU/cm^2^). The TT × Group interaction is also shown.

Term	Coefficient	Std. Error	T Stat	*p*-Value	95% CI Lower	95% CI Upper
**Intercept**	7.12	0.20	36.07	<0.0001	6.73	7.52
**TT (h)**	−0.42	0.01	−30.41	<0.0001	−0.45	−0.39
**Group**	−0.05	0.12	−0.44	0.66	−0.29	0.19
**TT × group**	−0.03	0.03	−1.15	0.26	−0.08	0.02

**Table 2:** Summary of logistic regression parameters and diagnostic performance metrics comparing the sensor-based method to the ISO 4833-2:2013 reference method. The table includes model coefficients (α, β), calculated detection limits (LOD_50_), relative limit of detection (RLOD), and performance indicators such as sensitivity, relative trueness, and false positive rate.

Category	Metric	Value
**Logistic regression**	α (Sensor)	−253.81
	β (Sensor)	94.43
	LOD_50_ (Sensor) [log CFU/cm^2^]	2.69
	α (ISO)	−751.64
	β (ISO)	306.94
	LOD_50_ (ISO) [log CFU/cm^2^]	2.45
	Relative Limit of Detection (RLOD)	1.10
**Performance metrics**	Sensitivity (Sensor)	0.93
	Repeatability (RSD %)	4.30
	Sensitivity (ISO vs. Sensor)	1.00
	Relative Trueness (RT)	0.94
	False Positive Rate (Sensor)	0.00

**Table 3:** Results of surface sampling in a meat processing environment using the swab-sachet sensor system. TT values determined at a ΔΦ threshold of 25° were converted to TVC (logCFU/cm^2^) using the calibration model (Equations (3) and (5)). Samples include both post-cleaning and in-processing conditions. <LOD indicates values below the limit of detection; nd: not detected.

Sample ID	Description	Sampling Time (hh:mm)	TT_25°_ (h)	TVC_sensor_ (logCFU/cm^2^) [EC Calibration] ^1^	TVC_sensor_ (logCFU/cm^2^) [Polymicrobial Calibration] ^2^
**Swab 1**	Teflon table—vacuum station ^3^	06:15	>12	<LOD	<LOD
**Swab 2**	Stainless steel tray—preparation room	06:25	11.65	0.09	1.02
**Swab 3**	Teflon table—vacuum station	10:15	10.91	0.47	1.34
**Swab 4**	Stainless steel tray—preparation room ^3^	10:15	>12	<LOD	<LOD
**Swab 6**	Operator gloves—dressing/cutting room (bovine) ^3^	06:15	>12	<LOD	<LOD
**SWAB 7**	Trolley—cutting room (bovine) ^3^	8:37	>12	<LOD	<LOD
**Swab 8**	Knife—dressing/cutting room (bovine)	10:25	0.55	5.75	5.79
**Swab 9**	Operator gloves—dressing/cutting room (bovine)	10:35	0.22	5.92	5.94
**SWAB 10**	Cutting room (bovine)	8:15	7.45	2.23	2.83
**SWAB 11**	Knife—dressing/cutting room (ovine)	8:17	2.12	4.95	5.12
**SWAB 12**	Trolley, cutting room	8:10	5.52	3.21	3.66
**SWAB 13**	Cutting room (ovine)	6:32	3.25	4.37	4.63

^1^ TVCsensor (logCFU/cm^2^) = −0.51TT(h) + 6.03 (Equation (3)), and ^2^ TVCsensor (logCFU/cm^2^) = −0.43TT(h) + 6.03 (Equation (6)); ^3^ surface sampled after cleaning.

**Table 4:** Step-by-step comparison between the conventional ISO 4833-2 reference method and the novel sensor swab-sachet method for surface TVC assessment. The table highlights the workflow steps, procedural differences, and estimated time required for each method. The sensor-based approach offers a simplified, faster, and less labour-intensive alternative with real-time monitoring capabilities, reducing the total time to results from 48 to 72 h to under 12 h.

Step	ISO 4833-2 Method (Reference)	Sensor Swab Sachet Method	Estimated Time (ISO)	Estimated Time (Sensor Swab Sachets)
**1. Surface Preparation**	Prepare 100 cm^2^ test surfaces		~10 min	
**2. Swab Sampling**	Swab area with pre-moistened sponge using 4-pass pattern		~2 min per sample	
**3. Sample Transfer**	Place swab into Stomacher bag with 100 mL BPW	Insert swab into sensor sachet pre-filled with 10 mL nutrient broth	~1 min	
**4. Homogenization/Sealing**	Homogenize in Stomacher	Heat-seal the sachet for airtight conditions	~2 min	30 s
**5. Serial Dilution/Sponge Activation**	Perform serial dilutions in BPW	Press sponge gently with pestle	~10 min	30 s
**6. Plating/Initial Measurement**	Plate on PCA using spread technique	Take first sensor reading	~10–15 min	5 s
**7. Incubation**	Incubate plates at 30 °C for 48–72 h	Incubate sachets at 30 °C	48–72 h	Up to 10 h
**8. Enumeration/Monitoring**	Manual CFU counting and calculation (post incubation)	Hourly sensor readings (3 replicates/sachet, during incubation)	~15 min/sample	~30 s

## Data Availability

The datasets presented in this article are not readily available because the data are part of an ongoing study. Requests to access the datasets should be directed to the corresponding author.

## Supplementary Materials

The following supporting information can be downloaded at: https://www.mdpi.com/article/10.3390/bios16090503/s1, Figure S1. Baseline analysis; Figure S2. ANCOVA diagnostics and calibration curve comparison between *E. coli* and polymicrobial samples; Table S1. Description of sources used for contaminating test surfaces with sample ID. Table S2. Estimated cost comparison between the proposed sensor-based method and standard plate counting for 1000 samples. The sensor approach shows comparable or lower cost per sample, with reduced labour requirements and faster time-to-result.

## References

[1] Yin W., Wang Y., Liu L., He J. (2019). Biofilms: The Microbial “Protective Clothing” in Extreme Environments. Int. J. Mol. Sci..

[2] Bai X., Nakatsu C.H., Bhunia A.K. (2021). Bacterial Biofilms and Their Implications in Pathogenesis and Food Safety. Foods.

[3] Gunduz G.T., Tuncel G. (2006). Biofilm Formation in an Ice Cream Plant. Antonie Leeuwenhoek Int. J. Gen. Mol. Microbiol..

[4] Van Houdt R., Michiels C.W. (2010). Biofilm Formation and the Food Industry, a Focus on the Bacterial Outer Surface. J. Appl. Microbiol..

[5] Liu X., Yao H., Zhao X., Ge C. (2023). Biofilm Formation and Control of Foodborne Pathogenic Bacteria. Molecules.

[6] Ban-Cucerzan A., Imre K., Morar A., Marcu A., Hotea I., Popa S.A., Pătrînjan R.T., Bucur I.M., Gașpar C., Plotuna A.M. (2025). Persistent Threats: A Comprehensive Review of Biofilm Formation, Control, and Economic Implications in Food Processing Environments. Microorganisms.

[7] Coughlan L.M., Cotter P.D., Hill C., Alvarez-Ordóñez A. (2016). New Weapons to Fight Old Enemies: Novel Strategies for the (Bio)Control of Bacterial Biofilms in the Food Industry. Front. Microbiol..

[8] Finn L., Onyeaka H., O’Neill S. (2023). Listeria Monocytogenes Biofilms in Food-Associated Environments: A Persistent Enigma. Foods.

[9] Bintsis T., Bintsis T. (2017). Foodborne Pathogens. AIMS Microbiol..

[10] Regulation—852/2004—EN—EUR-Lex. https://eur-lex.europa.eu/eli/reg/2004/852/oj/eng.

[11] Losito P., Visciano P., Genualdo M., Satalino R., Migailo M., Ostuni A., Luisi A., Cardone G. (2017). Evaluation of Hygienic Conditions of Food Contact Surfaces in Retail Outlets: Six Years of Monitoring. LWT.

[12] Moore G., Griffith C. (2007). Problems Associated with Traditional Hygiene Swabbing: The Need for in-House Standardization. J. Appl. Microbiol..

[13] Lewis T., Griffith C., Gallo M., Weinbren M. (2008). A Modified ATP Benchmark for Evaluating the Cleaning of Some Hospital Environmental Surfaces. J. Hosp. Infect..

[14] Griffith C. (2016). Surface Sampling and the Detection of Contamination. Handbook of Hygiene Control in the Food Industry.

[15] Querido M.M., Aguiar L., Neves P., Pereira C.C., Teixeira J.P. (2019). Self-Disinfecting Surfaces and Infection Control. Colloids Surf. B Biointerfaces.

[16] (2018). Microbiology of the Food Chain—Horizontal Methods for Surface Sampling.

[17] KovačEvić J., Bohaychuk V.M., Barrios R., Gensler G.E., Rolheiser D.L., Mcmullen L.M. (2009). Evaluation of Environmental Sampling Methods and Rapid Detection Assays for Recovery and Identification of *Listeria* Spp. from Meat Processing Facilities. J. Food Prot..

[18] Brauge T., Barre L., Leleu G., André S., Denis C., Hanin A., Frémaux B., Guilbaud M., Herry J.M., Oulahal N. (2020). European Survey and Evaluation of Sampling Methods Recommended by the Standard EN ISO 18593 for the Detection of Listeria Monocytogenes and Pseudomonas Fluorescens on Industrial Surfaces. FEMS Microbiol. Lett..

[19] (2013). Microbiology of the Food Chain—Horizontal Method for the Enumeration of Microorganisms—Part 2: Colony Count at 30 °C by the Surface Plating Technique.

[20] (2013). Microbiology of the Food Chain—Horizontal Method for the Enumeration of Microorganisms—Part 1: Colony Count at 30 Degrees C by the Pour Plate Technique.

[21] Robinson R.K. (2014). Encyclopedia of Food Microbiology.

[22] Santovito E., Elisseeva S., Bukulin A., Kerry J.P., Papkovsky D.B. (2021). Facile Biosensor-Based System for on-Site Quantification of Total Viable Counts in Food and Environmental Swabs. Biosens. Bioelectron..

[23] Abdallah A., Elisseeva S., Pinto L., Baruzzi F., Papkovsky D.B., Santovito E. (2025). Respirometric Sensor Vials for Rapid and Selective Detection of Coliforms in Raw Milk. Sens. Actuators B Chem..

[24] Soares A.R., Afonso R., Martins V.C., Palos C., Pereira P., Caetano D.M., Carta D., Cardoso S. (2022). On-Site Magnetic Screening Tool for Rapid Detection of Hospital Bacterial Infections: Clinical Study with Klebsiella Pneumoniae Cells. Biosens. Bioelectron. X.

[25] Petrucci S., Costa C., Broyles D., Dikici E., Daunert S., Deo S. (2021). On-Site Detection of Food and Waterborne Bacteria—Current Technologies, Challenges, and Future Directions. Trends Food Sci. Technol..

[26] Rampersad S.N. (2012). Multiple Applications of Alamar Blue as an Indicator of Metabolic Function and Cellular Health in Cell Viability Bioassays. Sensors.

[27] Elshikh M., Ahmed S., Funston S., Dunlop P., McGaw M., Marchant R., Banat I.M. (2016). Resazurin-Based 96-Well Plate Microdilution Method for the Determination of Minimum Inhibitory Concentration of Biosurfactants. Biotechnol. Lett..

[28] 5210 BIOCHEMICAL OXYGEN DEMAND (BOD)—Standard Methods for the Examination of Water and Wastewater. https://www.standardmethods.org/doi/10.2105/SMWW.2882.102.

[29] Benes O., Spanjers H., Holba M. (2002). Respirometry Techniques and Activated Sludge Models. Water Sci. Technol..

[30] Zhou H., Li D., Lee C., Zhou H., Li D., Lee C. (2025). Technology Landscape Review of In-Sensor Photonic Intelligence: From Optical Sensors to Smart Devices. AI Sens..

[31] Santovito E., Elisseeva S., Kerry J.P., Papkovsky D.B. (2023). Rapid Detection of Bacterial Load in Food Samples Using Disposable Respirometric Sensor Sachets. Sens. Actuators B Chem..

[32] Papkovsky D., Santovito E., Elisseeva S., Kerry J.P. (2022). Rapid Detection of Bacterial Load in Food Samples Using Disposable Respirometric Biosensor Sachets. SSRN.

[33] Santovito E., Elisseeva S., Cruz-Romero M.C., Duffy G., Kerry J.P., Papkovsky D.B. (2021). A Simple Sensor System for Onsite Monitoring of O_2_ in Vacuum-Packed Meats during the Shelf Life. Sensors.

[34] Li L., Gaspar R.D.L., Papkovsky D.B. (2024). Facile Barometric Calibration of Photoluminescence-Based Oxygen Sensors on a Standard Rotary Evaporator System with a Digital Vacuum Pump. Sens. Actuators B Chem..

[35] Cruz-Romero M.C., Santovito E., Kerry J.P., Papkovsky D. (2020). Oxygen Sensors for Food Packaging. Innovative Food Processing Technologies: A Comprehensive Review.

[36] Elisseeva S., Santovito E., Linehan E., Kerry J.P., Papkovsky D.B. (2023). Performance assessment of the two oxygen sensor based respirometric platforms with complex media and in selective bacterial assays. Sens. Actuators B Chem..

[37] O’Riordan T.C., Buckley D., Ogurtsov V., O’Connor R., Papkovsky D.B. (2000). A Cell Viability Assay Based on Monitoring Respiration by Optical Oxygen Sensing. Anal. Biochem..

[38] Goverde M., Willrodt J., Staerk A. (2017). Evaluation of the Recovery Rate of Different Swabs for Microbial Environmental Monitoring. PDA J. Pharm. Sci. Technol..

[39] Wang J., Guo X. (2024). The Gompertz Model and Its Applications in Microbial Growth and Bioproduction Kinetics: Past, Present and Future. Biotechnol. Adv..

[40] Shewhart W.A. (1931). Economic Control of Quality of Manufactured Product.

[41] Montgomery D.C., John Wiley & Sons (2019). Introduction to Statistical Quality Control.

[42] Doğan N.Ö. (2018). Bland-Altman Analysis: A Paradigm to Understand Correlation and Agreement. Turk. J. Emerg. Med..

[43] Zou G.Y. (2013). Confidence Interval Estimation for the Bland-Altman Limits of Agreement with Multiple Observations per Individual. Stat. Methods Med. Res..

[44] Giavarina D. (2015). Understanding Bland Altman Analysis. Biochem. Med..

[45] Bland J.M., Altman D. (1986). Statistical Methods for Assessing Agreement between Two Methods of Clinical Measurement. Lancet.

[46] Lakowicz J.R., Lakowicz J.R. (2006). Mechanisms and Dynamics of Fluorescence Quenching. Principles of Fluorescence Spectroscopy.

[47] Papkovsky D.B., Dmitriev R.I. (2013). Biological Detection by Optical Oxygen Sensing. Chem. Soc. Rev..

[48] Papkovsky D.B., Dmitriev R.I. (2018). Imaging of Oxygen and Hypoxia in Cell and Tissue Samples. Cell. Mol. Life Sci..

[49] Von Neumann J., Kent R.H., Bellinson H.R., Hart B.I. (1941). The Mean Square Successive Difference. Ann. Math. Stat..

[50] Santovito E., Elisseeva S., Smyth C., Cruz-Romero M., Kerry J.P., Duffy G., Papkovsky D.B. (2022). A Sensor-Based System for Rapid on-Site Testing of Microbial Contamination in Meat Samples and Carcasses. J. Appl. Microbiol..

[51] Garcia-Ochoa F., Gomez E., Santos V.E., Merchuk J.C. (2010). Oxygen Uptake Rate in Microbial Processes: An Overview. Biochem. Eng. J..

[52] Jarvis B. (2008). Statistical Aspects of the Microbiological Examination of Foods.

[53] Tuffley E.J., Lestang S.d., How J., Langlois T. (2018). Methodological Comparison for Sampling Populations of a Commercially Important Rock Lobster Species. Bull. Mar. Sci..

[54] Haghayegh S., Kang H.A., Khoshnevis S., Smolensky M.H., Smolensky M.H., Diller K.R. (2020). A Comprehensive Guideline for Bland-Altman and Intra Class Correlation Calculations to Properly Compare Two Methods of Measurement and Interpret Findings. Physiol. Meas..

[55] Cuny C., Lesbats M., Dukan S. (2006). Induction of a Global Stress Response during the First Step of Escherichia Coli Plate Growth. Appl. Environ. Microbiol..

[56] Elisseeva S., Bastiaanssen T.F.S., Santovito E., Zhdanov A.V., Cryan J.F., Kerry J.P., Papkovsky D.B. (2023). Combining the Oxygen Sensor Based Respirometry and 16S RRNA Amplicon Sequencing for the Analysis of Microbiota in Commercial Mince Products. Meat Sci..

[57] (2016). Microbiology of the Food Chain—Method Validation—Part 2: Protocol for the Validation of Alternative (Proprietary) Methods Against a Reference Method.

[58] Wilrich C., Wilrich P.-T. (2009). Estimation of the POD Function and the LOD of a Qualitative Microbiological Measurement Method. J. AOAC Int..

[59] Mărgăritescu I., Wilrich P.-T. (2013). Determination of the Relative Level of Detection of a Qualitative Microbiological Measurement Method with Respect to a Reference Measurement Method Microbiological Methods. J. AOAC Int..

