# Supplementary material for: Rapid Bacterial Detection on Surfaces by Field-Deployable Respirometric Sensor Sachets
Source: Biosensors (Basel). 2026 Sep 8;16(9):503. doi: 10.3390/bios16090503 (PMC13604147; doi:10.3390/bios16090503)
Supplement: Supplementary file 1 [file biosensors-16-00503-s001.zip › biosensors-4425293-supplementary.pdf]

SUPPLEMENTAL MATERIAL

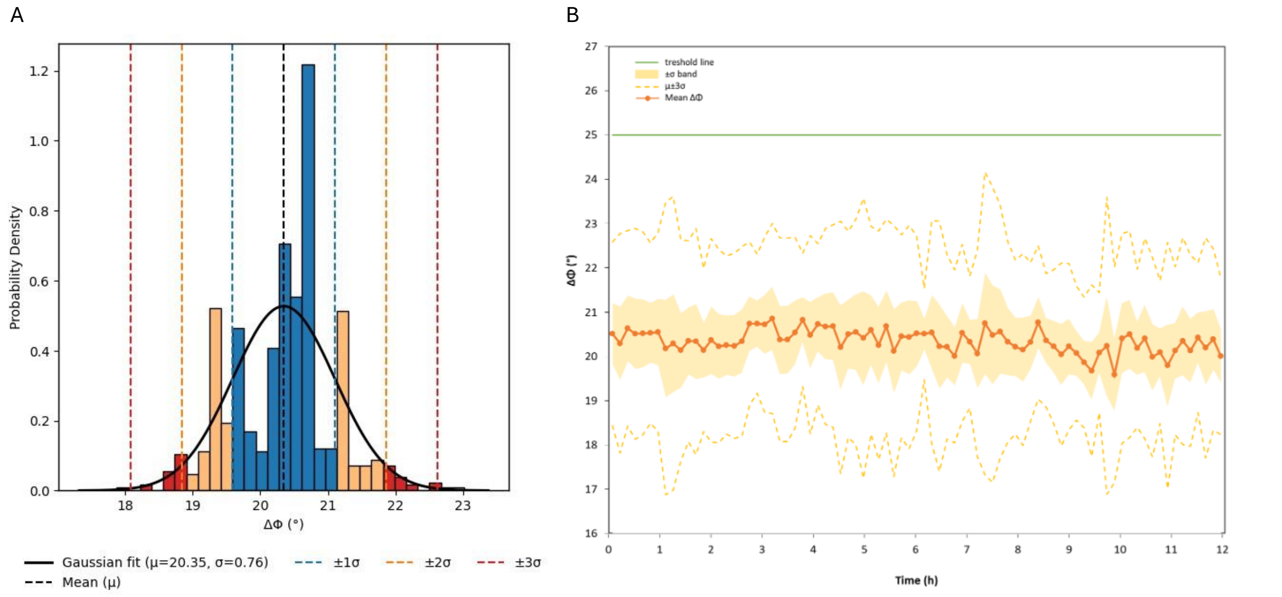

Figure S1. Baseline analysis. (A) Histogram of pooled baseline ΔΦ readings, with each bar coloured by its σ-region (blue = ±1σ, orange = ±2σ, red = ±3σ, gray = outside); overlaid is the fitted Gaussian curve (black), with dashed vertical lines marking the overall mean (black) and the ±1σ (blue), ±2σ (orange), and ±3σ (red) boundaries. (B) Mean phase-shift (ΔΦ) over time (orange line), with the shaded band showing ±1 σ variability across negative-control replicates (n=9). The dashed lines mark the statistical control limits at μ ± 3 σ (≈ 17.2° and 22.7°), and the solid green line at 25° denotes the practical QC threshold used for time-to-threshold determinations (no crossing occurred in these runs).

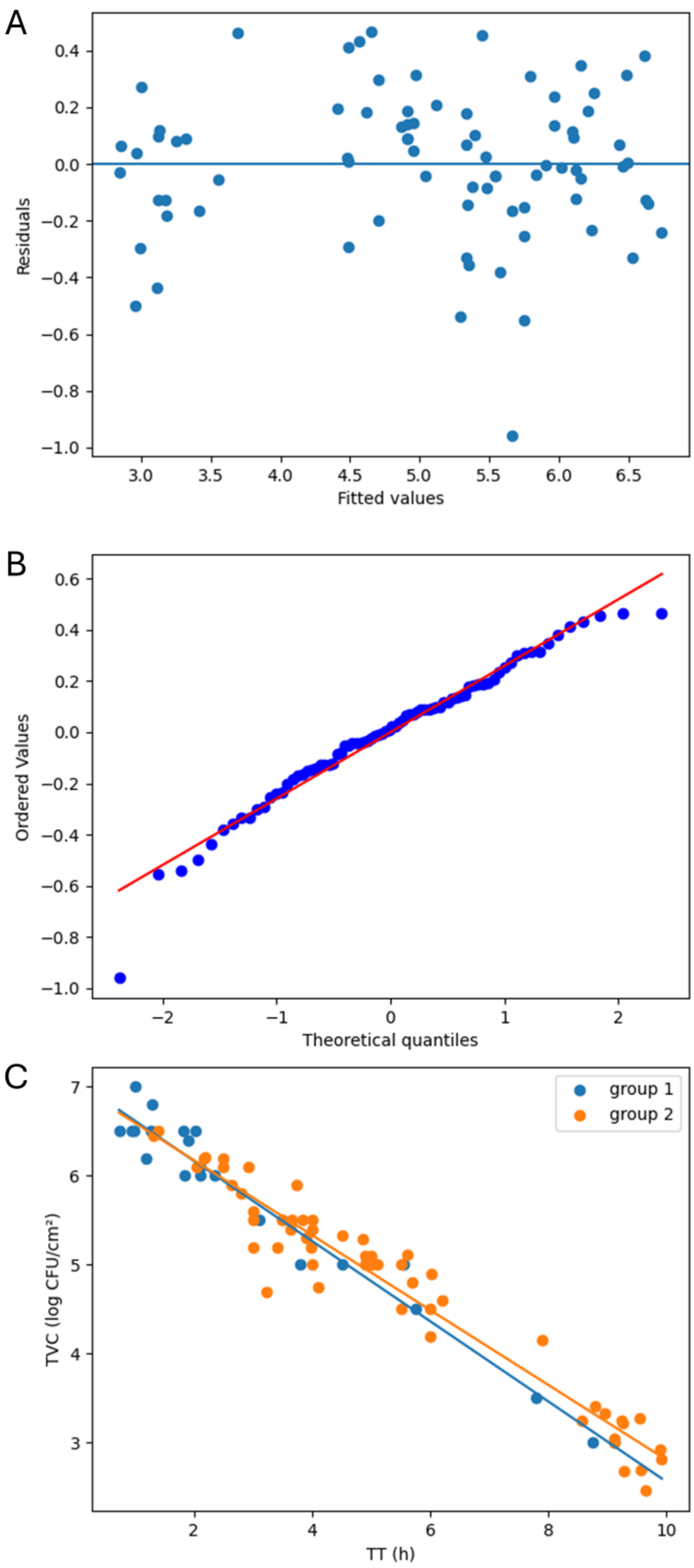

Figure S2. ANCOVA diagnostics and calibration curve comparison between E. coli and polymicrobial samples. Residual diagnostics and calibration curve comparison supporting the ANCOVA model. (A) Residuals versus fitted values plot showing no systematic pattern, indicating homoscedasticity. (B) Normal Q–Q plot of residuals, showing minor deviations from normality consistent with expected variability in biological datasets. (C) Calibration curves relating time-to-threshold (TT) to total viable count (TVC, log CFU/cm^2) for E. coli and polymicrobial samples. ANCOVA analysis confirmed no significant differences in slope (TT × group interaction, $p = 0.255$) or intercept (group effect, $p = 0.658$), supporting the use of a unified calibration model across sample types.

Table S1. Description of sources used for contaminating test surfaces with sample ID. The table reports the sample ID, inoculum type, and a brief description of each contamination source.

Sample ID	inoculum type	Description
**9.EC-1**	*E. coli*	Strain ATCC 8739 - day 1
**10.EC-1**	*E. coli*	Strain ATCC 8739 - day 1
**11.EC-1**	*E. coli*	Strain ATCC 8739 - day 1
**12.EC-1**	*E. coli*	Strain ATCC 8739 - day 1
**13.EC-2**	*E. coli*	Strain ATCC 8739 - day 2
**15.EC-2**	*E. coli*	Strain ATCC 8739 - day 2
**16.EC-2**	*E. coli*	Strain ATCC 8739 - day 2
**24.EC-2**	*E. coli*	Strain ATCC 8739 - day 2
**37.EC-3**	*E. coli*	Strain ATCC 8739 - day 3
**39.EC-3**	*E. coli*	Strain ATCC 8739 - day 3
**69.EC-3**	*E. coli*	Strain ATCC 8739 - day 3
**70.EC-3**	*E. coli*	Strain ATCC 8739 - day 3
**71.EC-4**	*E. coli*	Strain ATCC 8739 - day 4
**72.EC-4**	*E. coli*	Strain ATCC 8739 - day 4
**73.EC-4**	*E. coli*	Strain ATCC 8739 - day 4
**74.EC-4**	*E. coli*	Strain ATCC 8739 - day 4
**75.EC-5**	*E. coli*	Strain ATCC 8739 - day 5
**76.EC-5**	*E. coli*	Strain ATCC 8739 - day 5
**77.EC-5**	*E. coli*	Strain ATCC 8739 - day 5
**78.EC-5**	*E. coli*	Strain ATCC 8739 - day 5
**79.EC-6**	*E. coli*	Strain ATCC 8739 - day 6
**80.EC-6**	*E. coli*	Strain ATCC 8739 - day 6
**81.EC-6**	*E. coli*	Strain ATCC 8739 - day 6
**82.EC-6**	*E. coli*	Strain ATCC 8739 - day 6
**83.EC-6**	*E. coli*	Strain ATCC 8739 - day 6
**84.EC-6**	*E. coli*	Strain ATCC 8739 - day 6
**85.EC-7**	*E. coli*	Strain ATCC 8739 - day 7
**86.EC-7**	*E. coli*	Strain ATCC 8739 - day 7
**87.EC-8**	*E. coli*	Strain ATCC 8739 - day 8
**88.EC-8**	*E. coli*	Strain ATCC 8739 - day 8
**89.EC-8**	*E. coli*	Strain ATCC 8739 - day 8
**90.EC-8**	*E. coli*	Strain ATCC 8739 - day 8
**14.m-1**	meat	Beef steak (sirloin)
**17.m-2**	meat	Chicken breast (raw)
**18.m-3**	meat	Turkey thigh (skin-on)
**19.m-4**	meat	Chicken drumsticks
**20.m-5**	meat	Turkey thigh (skin-on)
**21.m-2**	meat	Chicken breast (raw)
**22.m-6**	meat	Pork loin
**23.m-7**	meat	Pork loin
**25.m-8**	meat	Chicken thigh (raw)

**26.m-9**	meat	Chicken wings
**27.m-10**	meat	Ground beef (butcherer-packaged)
**28.m-11**	meat	Ground beef (supermarket-packed)
**29.m-12**	meat	Beef chuck (for stew)
**30.m-13**	meat	Pork belly
**31.m-14**	meat	Beef chuck (for stew)
**32.m-15**	meat	Duck breast (raw)
**33.m-16**	meat	Ground turkey
**34.m-17**	meat	Poultry sausage (raw)
**35.m-18**	meat	Horse meat (steak)
**36.m-18**	meat	Horse meat (steak)
**38.m-19**	meat	Pork mince
**40.m-19**	meat	Pork mince
**41.m-4**	meat	Chicken drumsticks
**42.m-20**	meat	Turkey chops
**43.m-20**	meat	Turkey chops
**44.m-4**	meat	Chicken drumsticks
**1.v-1**	vegetables	Aubergine peel
**2.v-1**	vegetables	Aubergine peel
**3.v-2**	vegetables	Pepper (spoiled)
**4.v-2**	vegetables	Pepper
**5.v-3**	vegetables	Lettuce (unwashed)
**6.v-3**	vegetables	Lettuce (unwashed)
**7.v-4**	vegetables	Spinach (unwashed)
**8.v-5**	vegetables	Cauliflower (unwashed)
**45.v-6**	vegetables	Cucumbers (unwashed)
**46.v-7**	vegetables	Zucchini (spoiled)
**47.v-8**	vegetables	Wild Herbs
**48.v-9**	vegetables	Chicory
**49.v-10**	vegetables	Chard
**50.v-11**	vegetables	Turnip greens
**51.v-12**	vegetables	Arugula
**52.v-13**	vegetables	Leeks
**53.v-14**	vegetables	Spring onions
**54.v-15**	vegetables	Fennel
**55.v-16**	vegetables	Carrots
**56.v-17**	vegetables	Broccoli
**57.v-18**	vegetables	Celery
**58.v-19**	vegetables	Savoy cabbage
**59.v-20**	vegetables	Radicchio
**60.v-21**	vegetables	Asparagus
**61.v-22**	vegetables	Onion
**62.v-21**	vegetables	Asparagus (spoiled)

**63.v-4**	vegetables	Spinach (unwashed)
**64.v-23**	vegetables	Mixed salad (unwashed)
**65.v-23**	vegetables	Mixed salad (unwashed)
**66.v-22**	vegetables	Onion
**67.v-12**	vegetables	Arugula
**68.v-9**	vegetables	Chicory

Supplemental Table S2. Estimated cost comparison between the proposed sensor-based method and standard plate counting for 1000 samples. The sensor approach shows comparable or lower cost per sample, with reduced labour requirements and faster time-to-result

Cost component	***Sensor-based method (€)***	***Plate counting (ISO workflow) (€)***
Consumables	200	2000 – 4000
Sensor elements	5000	—
Reader (amortized)	250	—
Labor / operational costs	1000 – 2000	5000 – 10,000
Incubation / infrastructure	—	1000 – 2000
Total cost (1000 samples)	**6450 – 7450**	**8000 – 16,000**
Cost per sample (€)	**6.5 – 7.5**	**8 – 16**
